# Nutritional immunomodulation of Atlantic salmon response to *Renibacterium salmoninarum* bacterin

**DOI:** 10.3389/fmolb.2022.931548

**Published:** 2022-09-21

**Authors:** Mohamed Emam, Khalil Eslamloo, Albert Caballero-Solares, Evandro Kleber Lorenz, Xi Xue, Navaneethaiyer Umasuthan, Hajarooba Gnanagobal, Javier Santander, Richard G. Taylor, Rachel Balder, Christopher C. Parrish, Matthew L. Rise

**Affiliations:** ^1^ Department of Ocean Sciences, Memorial University of Newfoundland, St. John’s, NL, Canada; ^2^ Marine Microbial Pathogenesis and Vaccinology Laboratory, Department of Ocean Sciences, Memorial University of Newfoundland, St. John’s, NL, Canada; ^3^ Cargill Animal Nutrition and Health, Minneapolis, MN, United States

**Keywords:** *Salmo salar*, bacterial kidney disease, formalin-killed *Renibacterium salmoninarum* bacterin, qPCR, molecular biomarker, ω3 and ω6 dietary fatty acids

## Abstract

We investigated the immunomodulatory effect of varying levels of dietary ω6/ω3 fatty acids (FA) on Atlantic salmon (*Salmo salar*) antibacterial response. Two groups were fed either high-18:3ω3 or high-18:2ω6 FA diets for 8 weeks, and a third group was fed for 4 weeks on the high-18:2ω6 diet followed by 4 weeks on the high-18:3ω3 diet and termed “switched-diet”. Following the second 4 weeks of feeding (i.e., at 8 weeks), head kidney tissues from all groups were sampled for FA analysis. Fish were then intraperitoneally injected with either a formalin-killed *Renibacterium salmoninarum* bacterin (5 × 10^7^ cells mL^−1^) or phosphate-buffered saline (PBS control), and head kidney tissues for gene expression analysis were sampled at 24 h post-injection. FA analysis showed that the head kidney profile reflected the dietary FA, especially for C_18_ FAs. The qPCR analyses of twenty-three genes showed that both the high-ω6 and high-ω3 groups had significant bacterin-dependent induction of some transcripts involved in lipid metabolism (*ch25ha* and *lipe*), pathogen recognition (*clec12b* and *tlr5*), and immune effectors (*znrf1* and *cish*)*.* In contrast, these transcripts did not significantly respond to the bacterin in the “switched-diet” group. Concurrently, biomarkers encoding proteins with putative roles in biotic inflammatory response (*tnfrsf6b*) and dendritic cell maturation (*ccl13*) were upregulated, and a chemokine receptor (*cxcr1*) was downregulated with the bacterin injection regardless of the experimental diets. On the other hand, an inflammatory regulator biomarker, *bcl3*, was only significantly upregulated in the high-ω3 fed group, and a C-type lectin family member (*clec3a*) was only significantly downregulated in the switched-diet group with the bacterin injection (compared with diet-matched PBS-injected controls). Transcript fold-change (FC: bacterin/PBS) showed that *tlr5* was significantly over 2-fold higher in the high-18:2ω6 diet group compared with other diet groups. FC and FA associations highlighted the role of DGLA (20:3ω6; anti-inflammatory) and/or EPA (20:5ω3; anti-inflammatory) vs. ARA (20:4ω6; pro-inflammatory) as representative of the anti-inflammatory/pro-inflammatory balance between eicosanoid precursors. Also, the correlations revealed associations of FA proportions (% total FA) and FA ratios with several eicosanoid and immune receptor biomarkers (e.g., DGLA/ARA significant positive correlation with *pgds*, *5loxa*, *5loxb*, *tlr5*, and *cxcr1*). In summary, dietary FA profiles and/or regimens modulated the expression of some immune-relevant genes in Atlantic salmon injected with *R. salmoninarum* bacterin. The modulation of Atlantic salmon responses to bacterial pathogens and their associated antigens using high-ω6/high-ω3 diets warrants further investigation.

## 1 Introduction

Aquaculture is the fastest-growing food sector and one of the main contributors to the 2030 agenda for global nutrition security based on a recent report of the Food and Agriculture Organization of the United Nations (Hambrey, 2017; Stankus, 2021), notably considering the growing gap between animal protein production and increasing protein demand due to world population growth (Henchion et al., 2017). Atlantic salmon (*Salmo salar*) is one of the most economically important marine aquaculture species that shares in filling the gap between supply and human food demand (Asche et al., 2013). However, several nutritional and disease challenges threaten the Atlantic salmon aquaculture industry. There is growing evidence that nutritional modulations may be employed to improve Atlantic salmon’s immune response to the pathogens impacting its health and welfare (Caballero-Solares et al., 2017; Martin and Król, 2017; Katan et al., 2020; Xue et al., 2020).

Several studies have investigated the effects of dietary long-chain polyunsaturated fatty acids (LC-PUFA), for example, eicosapentaenoic acid (EPA) + docosahexaenoic acid (DHA), levels on Atlantic salmon immune responses (Martinez-Rubio et al., 2013, 2014; Arnemo et al., 2017; Bou et al., 2020; Xue et al., 2020). Also, dietary EPA and DHA levels modulated the transcript expression of genes with putative functions in innate immune response and oxidation-reduction homeostasis (Xue et al., 2020). In addition, EPA and DHA enhanced the production of anti-inflammatory cytokines and suppressed the expression levels of pro-inflammatory genes [e.g., *interleukin1b* (*il1b*)] in zebrafish (*Danio rerio*) (Cheng et al., 2015). However, the global limitation of fish oil (FO; currently the main EPA and DHA source for the aquafeed industry) resources remains a major challenge to increasing aquaculture production (Shepherd and Bachis, 2014; Sprague et al., 2016; Tocher et al., 2019). Terrestrial oil sources that have low EPA + DHA content are some of the current solutions for FO scarcity in the aquaculture industry (Sprague et al., 2017; Napier et al., 2020). However, the wide variety of vegetable oil (VO) fatty acid (FA) profiles, ranging from high-ω3 (e.g., 18:3ω3 as in linseed oil) to high-ω6 (e.g., 18:2ω6 as in soybean oil), with low EPA + DHA, adds another layer of challenge to incorporating those oil sources into aquaculture diets. The essential FAs that are prevalent in terrestrial oils (i.e., 18:3ω3 and 18:2ω6) and their elongation products play major structural and functional roles (e.g., membrane order, immune function, and lymphoid tissue development) in animals (Harbige, 2003; Arnemo et al., 2017; Bou et al., 2020; Hundal et al., 2020). Also, these FAs, and their desaturation and elongation products [e.g., ARA (20:4ω6; arachidonic acid), DGLA (20:3ω6; dihomo-gamma-linolenic acid), EPA, and DHA], are precursors for several eicosanoids mediating various inflammatory pathways (Bell et al., 1993, 1996). ARA (18:2ω6 desaturation and elongation product)-derived eicosanoids have pro-inflammatory roles (Calder, 2010), whereas DGLA (another desaturation and elongation product of 18:2ω6)-derived eicosanoids are considered anti-inflammatory (Kapoor and Huang, 2006; Baker et al., 2020). In addition, EPA and DHA-derived resolvins exhibit anti-inflammatory effects and can induce changes in gene expression to protect stimulated macrophages from excessive inflammation (Calder, 2010; Allam-Ndoul et al., 2017). Preponderantly, cells involved in inflammatory responses are rich in ω6-FA (e.g., ARA) (Calder, 2010); however, these FA profiles can be changed through dietary manipulation (Calder, 2010; Eslamloo et al., 2017). Thus, dietary FAs and consequently their derived eicosanoids are essential in regulating fish physiological and pathophysiological conditions.

Bacterial kidney disease (BKD) is one of the most widespread infectious diseases globally that can cause substantial economic losses for the salmon aquaculture industry (Rozas-Serri et al., 2020). *Renibacterium salmoninarum*, a fastidious Gram-positive bacterium and a member of the Micrococcaceae family (Fryer and Lannan, 1993), is the cause of BKD in salmonids. This bacterium can infect various salmonid species [e.g., sockeye salmon (*Oncorhynchus nerka*) and Atlantic salmon] in both fresh and marine water (Austin and Austin, 2016; Eslamloo et al., 2020a). Also, *R. salmoninarum* can be both horizontally and vertically transmitted (Hall et al., 2014). Antibiotic treatments are not fully effective for *R. salmoninarum* and can raise the antibiotic resistance threat (Austin, 1985; Rhodes et al., 2008; Fetherman et al., 2020). Also, vaccine efficacy to prevent BKD has been poor as the pathogen can survive for long periods in head kidney macrophages before being attacked by the immune system (Gu∂mundsdóttir et al., 2000; Grayson et al., 2002; Alcorn et al., 2005). Therefore, it is necessary to develop sustainable strategies such as dietary-based immunomodulation that can improve the Atlantic salmon’s resistance to this pathogen. In the present study, we tested if different diet formulations and regimens, hypothesized to be immunomodulatory, could change the response of Atlantic salmon to *R. salmoninarum*-derived antigens.

Providing low dietary EPA + DHA [0.3% used in (Emam et al., 2020) and 0.4% in the current study] alongside either high C_18_-ω3 (representing precursors of anti-inflammatory mediators) or high C_18_-ω6 (representing precursors of ARA as pro-inflammatory mediators) might promote selective elongation to the required LC-PUFA (e.g., DGLA, EPA, and DHA) from the dietary C_18_ precursors (Emam et al., 2020). Salmonids have the ability to elongate and desaturate FAs to fulfill their physiological needs (e.g., balance the pro-inflammatory and anti-inflammatory FA profile in the membranes) using the available dietary FAs (ω3 or ω6) (Leaver et al., 2008; Castro et al., 2012; Colombo et al., 2021). During immune stimulation (e.g., infection), a balance between pro- and anti-inflammatory responses of the host is required to minimize cellular damage caused by immune responses (Cicchese et al., 2018). Herein, we examined the effects of two diets formulated with 0.4% EPA + DHA and either high-ω3 or high-ω6 fed to salmon for 8 weeks, and a third group that was fed the high-ω6 diet for 4 weeks followed by 4 weeks of the high-ω3 diet (i.e., the “switched-diet” group) on the head kidney immune response. We used *R. salmoninarum* bacterin stimulation and previously microarray-identified, real-time quantitative polymerase chain reaction (qPCR)-confirmed *R. salmoninarum* bacterin-responsive biomarkers (Eslamloo et al., 2020b), together with other immune-relevant genes, to evaluate the antibacterial response in the head kidney of Atlantic salmon in the different dietary treatments.

## 2 Materials and methods

### 2.1 *R. salmoninarum* strain and bacterin preparation

*R. salmoninarum* ATCC33209 was cultured in KDM-2 [1.0% (w/v) peptone (Difco), 0.05% (w/v) yeast (Difco), 0.05% (w/v) L-cysteine HCl (Sigma-Aldrich, St. Louis, MO, United States), 10% (v/v) fetal bovine serum (Gibco, Thermo Fisher, Waltham, MA, United States), and 1.5% (v/v) *R. salmoninarum*-conditioned metabolite] (Evelyn et al., 1990) at 15°C under aerobic conditions. To prepare the bacterin, 1 L of KDM-2 was inoculated with 1 ml of fresh *R. salmoninarum* culture and grown at 15°C with aeration (180 rpm) for 10 days. The bacterial cells were harvested at an optical density (O.D. 600 nm) of 0.8 (∼1 × 10^8^ colony forming units ml^−1^) by centrifugation at 4,200 × *g* for 10 min at 4°C, washed three times with phosphate-buffered saline (PBS; pH 7.2, Gibco), and then inactivated using 6% formaldehyde (formalin; Sigma-Aldrich, Oakville, ON, Canada) with gentle agitation for 3 days at room temperature. Formalin was removed by centrifugation 4,200 × *g* for 10 min at 4°C. The bacterin was re-suspended and dialyzed in Slide-A-Lyzer Dialysis Cassettes (20 K MWCO, 12 ml, Thermo Fisher Scientific, Waltham, MA, United States) in PBS for 3 days at 6°C. Cell counting was performed using the Bacteria Counting Kit (Invitrogen, Thermo Fisher Scientific) by flow cytometry (BD FACS Aria ӀӀ flow cytometer, BD Biosciences, San Jose, CA, United States; using BD FACS Diva v7.0 software) following the manufacturer’s instructions. After confirming the bacterin inactivation using a subculture, the bacterin was stored at 4°C at a concentration of 10^8^ cells ml^−1^ and then diluted to a final concentration of 5 × 10^7^ cells ml^−1^ using PBS (Gibco; also used for the negative control injection) on the day of the immune challenge.

### 2.2 Feeding trial, immune challenge, and fish sampling

Two experimental diets were formulated to be isonitrogenous, isoenergetic (one with a high 18:3ω3 and the other with a high 18:2ω6; Table 1), and to meet the requirements of salmonids (NRC, 2011), with 0.4% dietary levels of EPA + DHA in both. Atlantic salmon smolts (initial body weight = 542.8 ± 122.65 g, mean ± standard deviation) were obtained from Cape d’Or Sustainable Seafood Inc. (Advocate Harbour, NS, Canada) and transferred to 38,000 L tanks at the Dr. Joe Brown Aquatic Research Building (Ocean Sciences Centre, Memorial University of Newfoundland, Canada). Fish were PIT (passive integrated transponder)-tagged and fed a standard commercial diet (EWOS Dynamic S, 5 mm, EWOS Canada, Surrey, BC, Canada) using automatic feeders (AVF6 Vibratory Feeder; Pentair Aquatic Eco-Systems, Inc., Nanaimo, BC, Canada) at a daily ration of 1% body weight. Feed rations were adjusted daily based on the number of uneaten pellets remaining from the previous feeding. In preparation for the feeding trial, smolts were randomly distributed into nine 620 L tanks (i.e., 26–27 fish per tank) and acclimated for 34 days. All tanks were connected to a flow-through filtered seawater system (12 L min^−1^; ∼12°C), and the photoperiod was 24 h light throughout the acclimation and experimental diet-testing periods.

**TABLE 1:** Formulation and fatty acid composition of the experimental diets fed to Atlantic salmon.

Ingredient (%)^a^	High-18:2ω6	High-18:3ω3
Fish meal	19.86	19.86
Plant protein^b^	49.45	49.45
Premix^c^	2.79	2.79
Fish oil	0.24	0.12
Soybean oil	21.00	-
Linseed oil	-	13.50
Poultry fat	6.64	14.26
Proximate composition (% as fed basis)		
Dry matter	96.57	96.86
Organic matter	94.00	93.94
Ash	5.80	5.87
Carbon	52.97	51.96
Hydrogen	8.78	8.69
Nitrogen	7.90	7.78
Fatty acid composition (% total FAs)^d^		
16:0	12.00	13.00
18:0	4.70	3.90
18:1ω9	22.00	23.00
18:2ω6; LNA	42.00	21.00
18:3ω3; ALA	5.00	27.00
20:5ω3; EPA	1.08	0.87
22:6ω3; DHA	1.40	1.20

a Experimental feeds were produced at the pilot plant at Cargill Innovation Center Dirdal, Norway.

b Plant protein: soy protein concentrate, wheat gluten, fava bean meal, pea protein concentrate, and raw wheat. For confidentiality, the nature and proportions of these plant products are not provided.

c Premix includes amino acids, vitamins, and pigment. Composition in micronutrients of the premix is proprietary information to Cargill, Inc.

d Only FAs >1.0% in at least one of the diets are included in the table.

DHA, docosahexaenoic acid; EPA, eicosapentaenoic acid.

**FIGURE 1:**
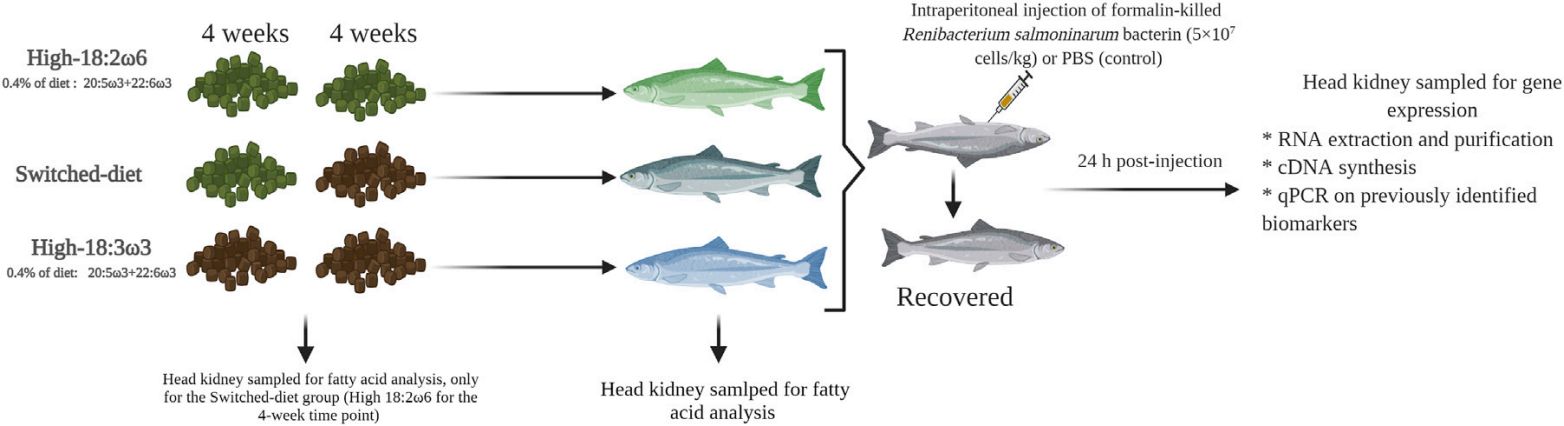
Experimental design showing sampling time points for fatty acid and qPCR analysis. This figure was constructed using https://biorender.com/.

At the beginning of the trial, three tanks were assigned to each dietary group [i.e., high-18:3ω3, high-18:2ω6, and switched-diet group]. The first group was fed a high 18:2ω6 diet [hypothesized to be a pro-inflammatory diet (Innes and Calder, 2018)], hereafter referred to as high-18:2ω6, for 8 weeks. The second group was fed a high 18:3ω3 diet [hypothesized to be an anti-inflammatory diet (Calder, 2010; Gutiérrez et al., 2019; Wen et al., 2019; Durkin et al., 2021)], hereafter referred to as high-18:3ω3, for 8 weeks. The third treatment (hereafter referred to as switched-diet) was fed for 4 weeks with high 18:2ω6 (same diet as high-18:2ω6) followed by 4 weeks with high 18:3ω3 FA (same diet as high-18:3ω3) (Figure 1). At the end of the feeding trial, fish were fasted for 24 h and euthanized using an overdose of MS-222 (400 mg L^−1^, Syndel Laboratories, Vancouver, BC, Canada). Then, fish fork length and body weight were recorded for 14–15 fish per tank for evaluating fish growth across the group as performance and health indicators. Head kidney samples (∼500 mg) were collected in 15-ml tubes containing 2 ml of chloroform and kept at −20°C for lipid analysis at 4 weeks in the switched-diet group and at 8 weeks for all groups. For the immune challenge, 6 fish per tank were lightly anesthetized (MS-222, 50 mg L^−1^), and three individuals per tank were subjected to intraperitoneal injection of *R. salmoninarum* bacterin (5 × 10^7^ cells kg^−1^; 1 ml kg^−1^ wet mass) as in the study by Eslamloo et al. (2020a). The remaining three fish in each tank were injected with PBS (1 ml kg^−1^ wet mass) as a sham-injection control. The injected fish were returned to the same tank, and anesthesia recovery was determined by observing active swimming. Fish were euthanized using MS-222 (400 mg L^−1^) 24 h post-injection, and head kidney samples were collected, flash-frozen in liquid nitrogen, and kept at −80°C for RNA-based studies. All procedures involving live fish were performed following the Canadian Council of Animal Care guidelines (Memorial University of Newfoundland Animal Care Protocol #18–04-MR).

### 2.3 Growth performance

Both condition factor (CF = weight/length^3^) and specific growth rate (SGR (% day^−1^) = 100 × [ln (final body weight)—ln (initial body weight)]/days) were used as performance and general health indicators (Morton and Routledge, 2006).

### 2.4 RNA extraction, purification, and cDNA synthesis

Total RNA was extracted from the head kidney tissues using TRIzol (Invitrogen/Life Technologies, Carlsbad, CA, United States) following manufacturer instructions. The head kidney samples were lysed in TRIzol using RNase-Free Disposable Pellet Pestles (Thermo Fisher Scientific) before RNA extraction. The RNA samples (40 µg of each) were treated with 6.8 Kunitz units of DNase (Qiagen, Mississauga, ON, Canada) for 10 min at room temperature following the manufacturer’s instructions. The DNase-treated RNAs were then purified using the RNeasy MinElute Cleanup kit (Qiagen) based on manufacturer recommendations (Xue et al., 2015; Caballero-Solares et al., 2017). The integrity and purity of purified RNA were checked using 1% agarose gel electrophoresis and NanoDrop spectrophotometry (NSW-1000), respectively. The RNA samples used in this study showed high purity (i.e., A260/280 and A260/230 ratios above 1.9 and 2.0, respectively) and integrity (tight 18S and 28S ribosomal RNA bands). One microgram of purified RNA was used for cDNA synthesis in a 20 µl reaction using random primers (250 ng; Invitrogen, Thermo Fisher Scientific), M-MLV reverse transcriptase (200 U; Invitrogen/Life Technologies), first-strand buffer (1× final concentration), dNTPs (0.5 mM final concentration), and DTT (10 mM final concentration) at 37°C for 50 min (Xue et al., 2015).

### 2.5 Real-time quantitative polymerase chain reaction analysis

The qPCR assays were performed using 384-well plates and a ViiA 7 Real-Time PCR system (Applied Biosystems, Thermo Fisher Scientific). Each qPCR reaction (13 µl in total) consisted of 1 × Power SYBR Green PCR Master Mix (Applied Biosystems, Thermo Fisher Scientific), 50 nM of each forward and reverse primer, and the indicated amount of cDNA (see below). All qPCR assays were carried out in triplicate in this study. The qPCR program (for all targeted genes) consisted of 1 cycle of 50°C for 2 min, 1 cycle of 95°C for 10 min, 40 cycles of 95°C for 15 s, and 60°C for 1 min, with fluorescence detection at the end of each 60°C step. The dissociation curve analysis was performed for each gene of interest (GOI) and normalizer gene (i.e., each primer pair) and showed a single product (i.e., a single peak). The primers used were either adopted from previously published studies (Caballero-Solares et al., 2017; Eslamloo et al., 2020b, 2020a) or newly designed to target bacteria recognition, lipid metabolism, and eicosanoid-relevant biomarker genes (Table 2). Primer design was performed using the online PrimerQuest^®^ tool (https://www.idtdna.com/Primerquest/Home/Index). Two separate pools of PBS and bacterin-injected groups were generated using the cDNA of the individuals [1 μg of input total RNA for each individual in the pool (i.e., equal representation of each individual in a given pool)] used in the qPCR study for primer quality control (QC) of the studied genes. Amplification efficiencies (Pfaffl, 2001) of the GOIs and normalizers were generated using a three-fold serial dilution (five-point) of a given cDNA (with a cDNA template representing 10 ng of input total RNA). To select the normalizer genes, seven candidate normalizers were tested using all samples from all the experimental groups, and the cycle threshold (C_T_) values were analyzed by geNorm (Vandesompele et al., 2002). Both *eukaryotic translation initiation factor 3 subunit D* [*eif3d*, (Caballero-Solares et al., 2017)] and *60S ribosomal protein 32* [*rpl32*, (Xue et al., 2015)] showed the most stable expression across all the samples and groups (M-value < 0.21). The mRNA levels of the GOIs were measured using a cDNA template representing 5 ng of input total RNA for each qPCR reaction. All GOIs and normalizers were tested using nine fish per condition (i.e., PBS and bacterin-injected fish in each dietary group) for the three treatments as well as a no-template control. The relative quantity (RQ) of each GOI was calculated using the qBase relative quantification framework for ΔΔC_T_ analysis (Livak and Schmittgen, 2001). This was performed using the C_T_ values for GOIs, normalized to both *eif3d* and *rpl32*, and incorporating the amplification efficiencies (Table 2). For each GOI, RQ values were calibrated to the individual with the lowest normalized transcript expression (i.e., RQ = 1.0) and presented as mean ± standard error (S.E). The RQ data were used to statistically compare the bacterin-injected with the PBS-injected within each diet group, including biological variability of transcript expression for both immune-stimulated and control fish. The fold-change (FC) data were used to determine trends across dietary treatments and associations with FAs.

**TABLE 2:** Primers used for qPCR.

Gene name	Gene symbol	GenBank accession number	F and R^a^	Primer sequence (5′–3′)	Efficiency (%)	Amplicon size (bp)	Reference
*arachidonate 12-lipoxygenase*	*12lox*	BT072280.1	F	GGG​TCA​GGA​CAG​AGT​TTA​GGA	91	142	^b^
			R	TGG​GCA​GTA​GGA​AGA​GGT​AAG			
*15-lipoxygenase b-like*	*15lox*	XM_014124184.2	F	CCA​CAA​TGG​GAG​CCA​GAA​TAC	94	96	^b^
			R	GGG​TTA​CAG​CCG​TTC​AAA​CA			
*arachidonate 5-lipoxygenase a*	*5loxa*	NM_001139832.1	F	CTG​CTC​ACC​ATG​CTG​CTG​TC	95	93	^b^
			R	GTGTGGGAGGAGGCTTCC			
*arachidonate 5-lipoxygenase b*	*5loxb*	CX354498	F	ACT​GCT​GTG​GGT​TTC​CCA​AG	103	98	^b^
			R	GAC​AGC​AGC​GTG​ATG​TGC​AG			
*b-cell lymphoma 3 protein-like*	*bcl3*	EG843167.1	F	CAC​ACC​AAC​ATC​CCT​TAC​CC	92	112	^b^
			R	CTT​TGC​TCG​TGA​TGG​AGA​CA			
*cc motif chemokine 13*	*ccl13*	BT048088	F	ACT​CCT​CCT​GGG​ACT​GCT​CT	95	109	Eslamloo et al. (2020b)
			R	CCT​CTT​TGG​GTG​GAA​CTT​CA			
*cholesterol 25-hydroxylase-like protein a*	*ch25ha*	BT046542	F	TAG​AGC​TGT​GAT​GCT​AGT​TTA​C	100	106	Eslamloo et al. (2020b)
			R	ACC​CAG​TAG​CAC​TGA​GAA​GTC			
*cytokine-inducible sh2-containing protein*	*cish*	BT057484	F	TGG​AGC​CAC​GTC​AGA​CAT​AA	108	153	Eslamloo et al. (2020b)
			R	GCA​CCA​TGT​GTT​TTC​CAG​TG			
*c-type lectin domain family 12-member b*	*clec12b*	EG842232	F	GGG​TAT​TGG​ATC​GGT​TTG​AC	106	109	Eslamloo et al. (2020b)
			R	TCC​CTC​CAT​TTC​GAC​TGT​TC			
*c-type lectin domain family 3-member a*	*clec3a*	EL698766.1	F	CCA​ACC​GTT​ACT​GGA​GCA​CT	97	174	Eslamloo et al. (2020b)
			R	GGC​TCC​CCT​TAA​CCC​AGA​TA			
*cyclooxygenase 1*	*cox1*	BT045745	F	CTC​ATG​AGG​GTG​GTC​CTC​AC	98	135	Caballero-Solares et al. (2017)
			R	AGG​CAC​AGG​GGG​TAG​GAT​AC			
*c-x-c chemokine receptor type 1-like*	*cxcr1*	CX355704	F	ATG​CTG​ATT​CCC​CCT​ACT​CC	97	103	Eslamloo et al. (2020b)
			R	ACA​CTG​CTC​AAG​CCC​AAG​AT			
*interferon regulatory factor 1*	*irf1*	BT048538	F	GCA​ATG​AAG​TAG​GCA​CAG​CA	94	100	Eslamloo et al. (2020a)
			R	CGC​AGC​TCT​ATT​TCC​GTT​TC			
*lipase e, hormone-sensitive*	*lipe*	NM_001140535	F	ACC​CAA​CTT​TCC​ACG​TCA​AG	95	137	Eslamloo et al. (2020b)
			R	CAG​TAG​ATC​CCC​GAT​GTC​GT			
*leukotriene a-4 hydrolase-like*	*lta4h*	XM_014152853	F	TGA​CAA​CGG​AGG​TGG​AAC​T	97	80	^b^
			R	ATG​GCA​ATG​TCC​GCT​TTA​GG			
*nf-kappa-b inhibitor alpha*	*nfkbia*	BT058522.1	F	TGG​ACC​TTC​ATC​GAA​CGA​GAA	94	97	^b^
			R	CGC​TCT​TCA​TTG​AAA​GAT​TTA​AC			
*lipocalin-type prostaglandin d synthase*	*pgds*	BT048787	F	GGT​GCT​CAA​CAA​GCT​CTA​CA	92	114	^b^
			R	GCA​GGA​AAG​CGA​TGT​TGT​CA			
*toll-like receptor 2*	*tlr2*	XM_045721418	F	CAC​CCG​TCT​GGA​CAA​ACT​AAT​C	109	102	^b^
			R	ATG​TTG​AGG​TGA​GTC​AGG​GT			
*toll-like receptor 5*	*tlr5*	AY628755	F	ATC​GCC​CTG​CAG​ATT​TTA​TG	102	101	Eslamloo et al. (2020b)
			R	GAG​CCC​TCA​GCG​AGT​TAA​AG			
*toll-like receptor 9*	*tlr9*	NM_001123653	F	AGA​CTC​CAG​TGT​GGT​GAA​CT	91	109	^b^
			R	TAC​CTG​CTG​AAT​CCC​GGT​ATA​G			
*tumor necrosis factor receptor superfamily member 11b*	*tnfr11b*	BT049358	F	CTG​TCC​TCA​GGG​GTA​CGT​GT	99	154	Eslamloo et al. (2020b)
			R	CTG​ACC​AGC​TTC​CTC​AGC​TT			
*tumor necrosis factor receptor superfamily member 6b*	*tnfrsf6b*	EG881931	F	CCC​AGG​TCG​CAC​CAC​TAT​AC	96	112	Eslamloo et al. (2020b)
			R	CAT​CAA​CTC​CCC​ATC​ACA​GA			
*e3 ubiquitin-protein ligase znrf1*	*znrf1*	EG922586	F	CAG​CAC​GTC​ATC​GTT​GTA​GG	92	103	Eslamloo et al. (2020b)
			R	CAA​GTG​TCC​TGT​CTG​CTC​CA			
*60s ribosomal protein 32*	*rpl32*	BT043656	F	AGG​CGG​TTT​AAG​GGT​CAG​AT	93	119	Xue et al. (2015)
			R	TCG​AGC​TCC​TTG​ATG​TTG​TG			
*eukaryotic translation initiation factor 3 subunit d*	*eif3d*	GE777139	F	CTC​CTC​CTC​CTC​GTC​CTC​TT	92	105	Caballero-Solares et al. (2017)
			R	GAC​CCC​AAC​AAG​CAA​GTG​AT			

a Forward (F) and Reverse (R) Primers.

b Primers designed in the current study.

### 2.6 Fatty acid analysis of head kidney tissues

We aimed to explore the FA profile of the head kidney tissues as one of the main immune organs and the same organ explored in our gene expression study. Lipid extraction and FA derivatization were performed as described (Katan et al., 2019; Emam et al., 2020). Briefly, all utensils were lipid-cleaned (three times with methanol followed by three times with chloroform or burned in the muffle furnace at 550°C for 6 h); the samples were kept on ice during the homogenization process, and they were covered with nitrogen after all steps. Chloroform: methanol: water (8: 4: 3) mixtures were used for the extraction process. The Hilditch reagent (1.5 H_2_SO_4_: 98.5 anhydrous MeOH) was used to derivatize FAs in 50 µl of the lipid extracts for 1 h at 100°C. Transesterified samples were analyzed in an HP 6890 gas chromatograph on a Zebron ZB-WAX plus™ (30 m × 0.32 mm × 0.25 µm) column (Zebron, Phenomenex, Aschaffenburg, Germany). Derivatized samples were injected at 65°C, and the temperature was increased at a rate of 40°C min^−1^ to 195°C and then increased to 220°C at a rate of 2°C min^−1^. The hydrogen carrier gas flow was 2 ml min^−1^, and the starting temperature of the injector was 150°C with an increase of 120°C min^−1^ to 250°C. The detector temperature was kept at 260°C. The obtained peaks were compared to those obtained using standards from Supelco (Bellefonte, PA, United States): 37 component FA methyl ester (FAME) mix (Product number 47885-U), PUFA 3 (product number 47085-U), and PUFA 1 (product number 47033-U). Chromatograms were integrated using Chromatography Data Systems Open Laboratory CDS, and the FA data were calculated as an area percent of FAME.

### 2.7 Statistical analysis

**FIGURE 2:**
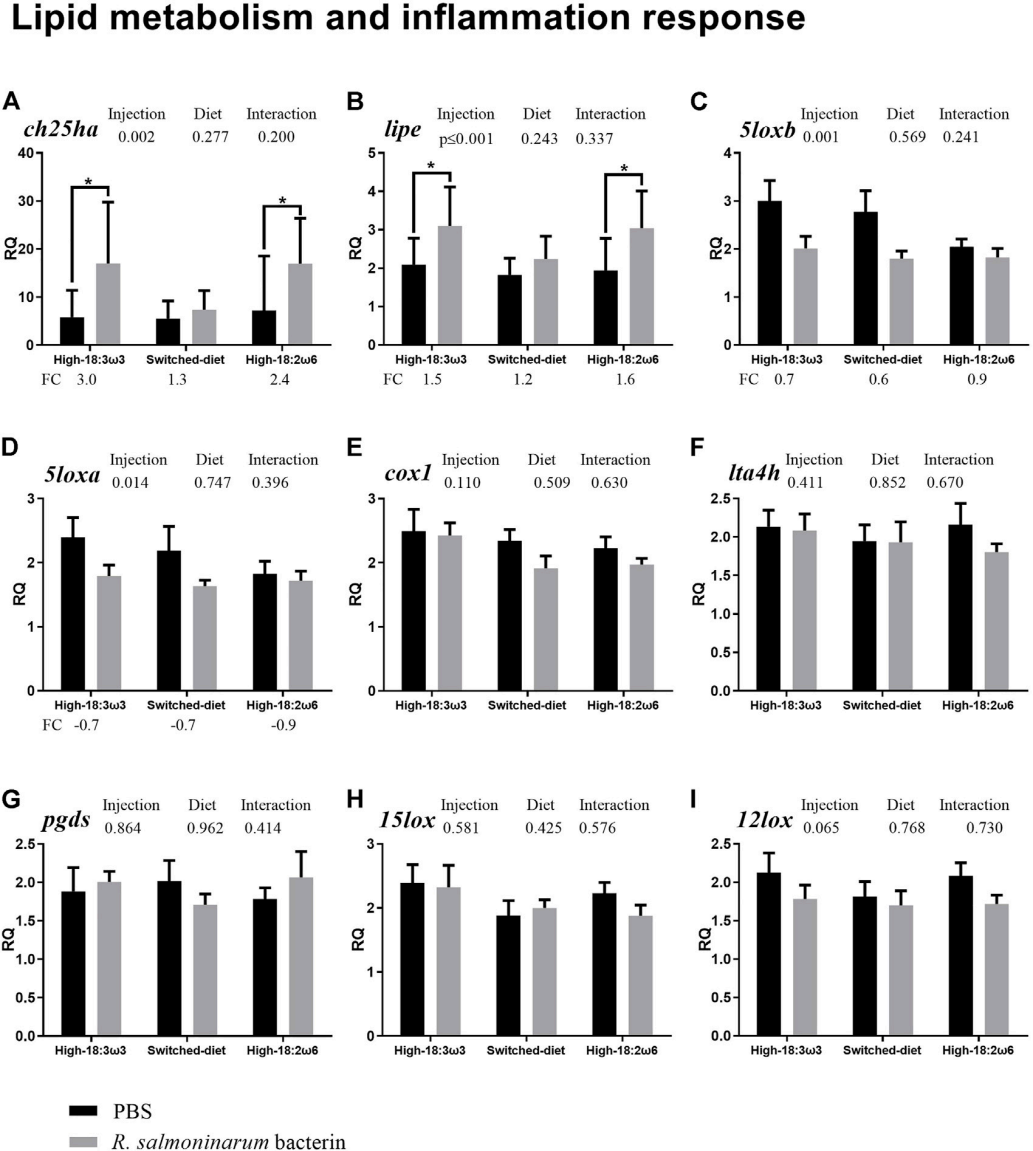
qPCR for transcripts playing roles related to lipid metabolism and inflammation (**(A–I)**; individual RQs used in the analysis *n* = 9). Two-way ANOVA results are shown in the table above each panel, and *t*-test results (i.e., significant differences between PBS and bacterin-stimulated fish within a diet group) are shown with asterisks (“*” for *p* ≤ 0.05). Data are presented as mean ± SE. Overall fold-change values (FC; mean RQ in bacterin-injected group/mean RQ in PBS-injected group) are shown below the transcripts with significant differences between groups. The significance threshold was adjusted to *p* ≤0.05. **(A)** *cholesterol 25-hydroxylase-like protein a*; **(B)** *lipase e, hormone-sensitive*; **(C)** *arachidonate 5-lipoxygenase b*; **(D)** *arachidonate 5-lipoxygenase a*; **(E)** *cyclooxygenase 1*; **(F)** *leukotriene a-4 hydrolase-like*; **(G)** *lipocalin-type prostaglandin d synthase*; **(H)** *15-lipoxygenase b-like*; **(I)** *arachidonate 12-lipoxygenase*.

**FIGURE 3:**
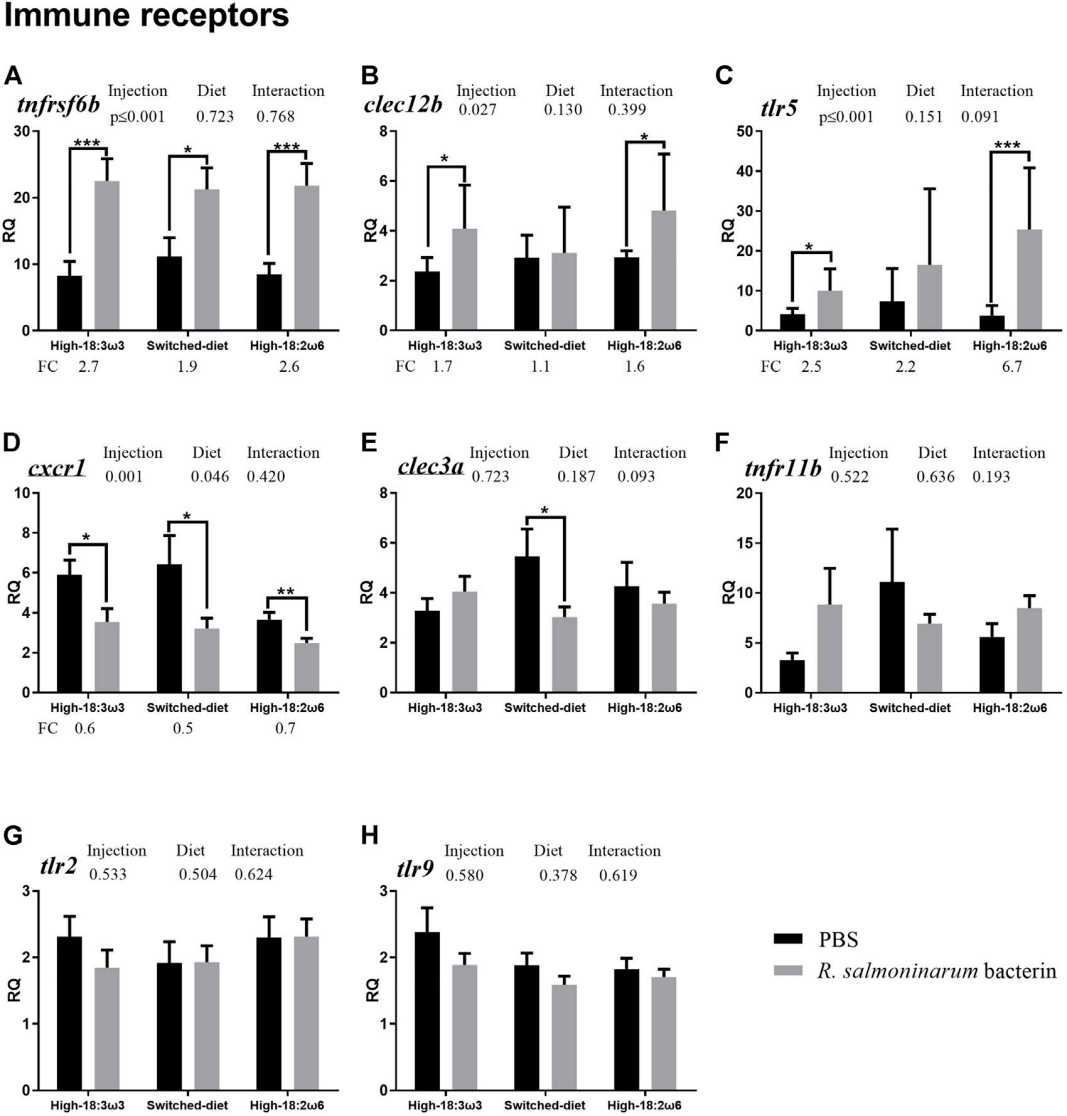
qPCR for transcripts representing the immune receptors (**(A–H)**; individual RQs used in the analysis *n* = 9). Two-way ANOVA results are shown in the table above each panel, and *t*-test results (i.e., significant differences between PBS and bacterin-stimulated fish within a diet group) are shown with asterisks (“*” for *p* ≤ 0.05, “**” for *p* ≤ 0.01, and “***” for *p* ≤ 0.001). Data are presented as mean ± SE. Overall fold-change values (FC; mean RQ in bacterin-injected group/mean RQ in PBS-injected group) are shown below the transcripts with significant differences between groups. The significance threshold was adjusted to *p* ≤ 0.05. **(A)** *tumor necrosis factor receptor superfamily member 6b*; **(B)** *c-type lectin domain family 12-member b*; **(C)** *toll-like receptor 5*; **(D)** *c-x-c chemokine receptor type 1-like*; **(E)** *c-type lectin domain family 3-member a*; **(F)** *tumor necrosis factor receptor superfamily member 11b*; **(G)** *toll-like receptor 2*; **(H)** *toll-like receptor 9*.

**FIGURE 4:**
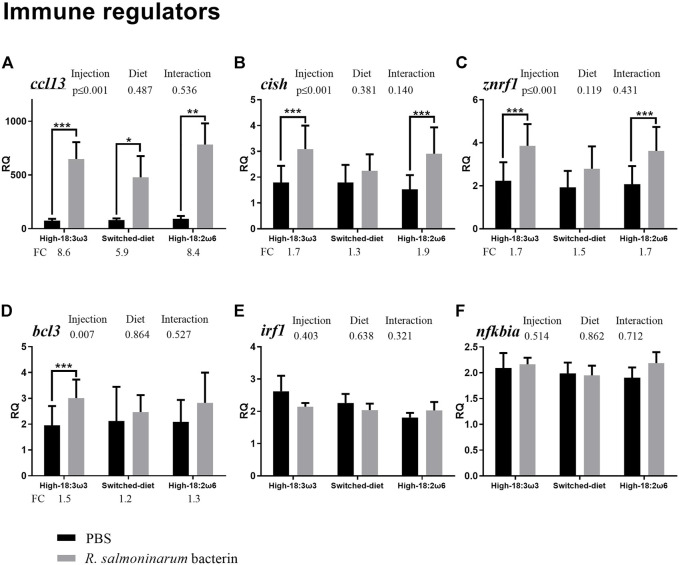
qPCR for transcripts representing the immune effectors and transcription factors (**(A–F)**; individual RQs used in the analysis *n* = 9). Two-way ANOVA results are shown in the table above each panel, and *t*-test results (i.e., significant differences between PBS and bacterin-stimulated fish within a diet group) are shown with asterisks (“*” for *p* ≤ 0.05, “**” for *p* ≤ 0.01, and “***” for *p* ≤ 0.001). Data are presented as mean ± SE. Overall fold-change values (FC; mean RQ in bacterin-injected group/mean RQ in PBS-injected group) are shown below the transcripts with significant differences between groups. The significance threshold was adjusted to *p* ≤ 0.05. **(A)** *cc motif chemokine 13*; **(B)** *cytokine-inducible sh2-containing protein*; **(C)** *e3 ubiquitin-protein ligase znrf1*; **(D)** *b-cell lymphoma 3 protein-like*; **(E)** *interferon regulatory factor 1*; **(F)** *nf-kappa-b inhibitor alpha*.

All residuals were examined for normality and homoscedasticity (i.e., Shapiro–Wilk and Levene’s tests, respectively). The *t*-test or Mann–Whitney test was used to analyze the bacterin injection effects (i.e., bacterin vs. PBS using RQs) within each dietary treatment independently for data with normal or non-normal distribution, respectively (Figures 2–4). One-way ANOVA followed by Tukey post-hoc tests was used to identify the difference between all groups (injection/diet; i.e., six groups) for normally distributed data (i.e., RQs and FC), whereas the Kruskal–Wallis test was used to determine significant differences among injection-matched dietary groups with non-normal distribution (Supplementary Table S1 and Supplementary Figure S1, respectively). Two-way ANOVA was used to explore the overall diet and the injection effects, and the interaction between them, using RQs. One-way ANOVA and *t*-test were used to compare FAs percent between groups at 8 weeks and comparing switched diet at 4 weeks vs. 8 weeks, respectively. Statistical analyses were performed using IBM SPSS (IBM SPSS Statistics, Version 25, Armonk, NY, United States) and SigmaPlot (Systat Software, San Jose, CA, United States). Tank means were used to explore associations between FAs and gene expression. Principal coordinates analysis (PCoA), permutational multivariate ANOVA (PERMANOVA), and SIMPER (similarity percentage) analysis were conducted for the standardized tank means of the targeted transcript FC (individual RQ of the bacterin-stimulated individual/average RQ of the PBS-injected fish for each group) and FAs (at more than 1% of total) using PRIMER 7 (PRIMER-E Ltd., Auckland, New Zealand) with r = 0.2. Also, tank means were used to explore the correlation between FAs and FC for each GOI using IBM SPSS. The transcript FC associations were explored with Pearson’s correlation in the “corrplot” package in R; the significance level was adjusted to *p* ≤ 0.05. All the RQs were standardized and then subjected to PCoA, PERMANOVA, and SIMPER, and then the results were plotted and summarized in Supplementary Figure S2. Also, the FAs were subjected to PCoA analysis using the individual fish (Supplementary Figures S3A, S3B).

## 3 Results

### 3.1 Growth performance

Dietary treatments did not significantly affect either the CF or SGR (high-18:3ω3: 1.19 ± 0.02 and 0.77 ± 0.03; switched-diet: 1.19 ± 0.02 and 0.77 ± 0.04; high-18:2ω6: 1.27 ± 0.04 and 0.79 ± 0.05, means ± standard errors, *p*-values = 0.15 and 0.86, respectively).

### 3.2 Gene expression of Atlantic salmon head kidney in response to *R. salmoninarum* bacterin

The qPCR results showed that the bacterin IP injection significantly upregulated the transcript levels of *cholesterol 25-hydroxylase-like protein a* (*ch25ha*) and *lipase e, hormone-sensitive* (*lipe*) when compared with the PBS-injected fish in both the high-18:3ω3 and high-18:2ω6 groups but not in the switched-diet group (Figures 2A, B; Supplementary Table S1). The transcript levels of *arachidonate 5-lipoxygenase b* (*5loxb*) were higher in the PBS-injected high-18:3ω3 group than in the PBS-injected high-18:2ω6 group, but no significant differences in this gene were seen among diet groups for bacterin-stimulated fish (Supplementary Table S1; one-way ANOVA). Also, there was significant downregulation of *5loxb* with the bacterin injection in both the high-18:3ω3 and the switched-diet groups when compared with the diet-matched PBS-injected group (Figure 2C). The factor “injection” was significant using two-way ANOVA (shown above each bar plot) for two genes upregulated by bacterin (*ch25ha* and *lipe*) and two genes downregulated by bacterin (*5loxa* and *5loxb*) (Figures 2A–D). There was no significant interaction between diet and injection across the investigated genes. The levels of *cyclooxygenas*e *1* (*cox1*), *leukotriene A-4 hydrolase-like* (*lta4h*), *lipocalin-type prostaglandin d synthase* (*pgds*), *arachidonate 12-lipoxygenase* (*12lox*), and *15-lipoxygenase B-like* (*15lox*) did not show significant differences across the experimental groups (Figures 2E–I).

As shown in Figure 3 (i.e., immune receptor-related transcripts), the levels of *tumor necrosis factor receptor superfamily member 6b* (*tnfrsf6b*) were significantly higher in the bacterin-injected groups than in diet-matched PBS controls for all three diet regimens; however, the induction in the switched-diet group (1.9-fold) was somewhat lower than that in the other two diet groups (2.6–2.7-fold), with a difference in the significance level as well (Figure 3A). The bacterin injection significantly upregulated the expression of *C-type lectin domain family 12-member b* (*clec12b*) and *toll-like receptor 5* (*tlr5*) when compared with the PBS-injected fish in the high-18:3ω3 and high-18:2ω6 groups but not in the switched-diet group (Figures 3B, C). The transcript expression of *tlr5* was significantly higher in bacterin-stimulated fish fed the high-18:2ω6 diet than in bacterin-stimulated fish fed the high-18:3ω3 diet; also, *tlr5* upregulation by the bacterin was over two-fold significantly higher in the high-18:2ω6 diet fed fish (i.e., 6.7-fold) than in the other two diet groups (i.e., 2.2–2.5-fold) compared with diet-matched PBS controls (Figure 3C and Supplementary Figure S1). Although *c-x-c chemokine receptor type 1-like* (*cxcr1*) was significantly downregulated by the bacterin in all dietary treatments (Figure 3D), the bacterin-dependent suppression of *c-type lectin domain family 3-member a* (*clec3a*) only occurred in the switched-diet group (Figure 3E). The two-way ANOVA showed the factor “injection” was significant for three genes upregulated by bacterin stimulation (*tnfrsf6b*, *clec12b*, and *tlr5*) and one gene downregulated by bacterin (*cxcr1*) (Figures 3A–D). There was no significant interaction between diet and injection across the investigated genes. The levels of *tumor necrosis factor receptor superfamily member 11b* (*tnfr11b*), *toll-like receptor 2* (*tlr2*), and *toll-like receptor 9* (*tlr9*) were not different across groups (Figures 3F–H).

As shown in Figure 4 (immune effectors and transcription factors), the transcript levels of *CC motif chemokine 13* (*ccl13*) were significantly higher in the bacterin-injected fish than in the PBS-injected fish in all three dietary groups; however, the induction in the switched-diet group (∼5.9-fold) was somewhat lower, and with a lower significance level, than that in the other two diet groups (∼8.4–8.6-fold) (Figure 4A). Also, the bacterin IP injection significantly upregulated the transcript levels of *cytokine-inducible sh2-containing protein* (*cish*) and *E3 ubiquitin-protein ligase* (*znrf1* alias *zinc/RING finger protein 1*) when compared with the PBS-injected fish in both high-18:3ω3 and high-18:2ω6 diet-fed groups but not in the switched-diet group (Figures 4B, C). The bacterin injection significantly upregulated *b-cell lymphoma 3 protein-like* (*bcl3*) only in the high-18:3ω3 group when compared with the diet-matched PBS-injected controls (Figure 4D). The two-way ANOVA indicated the factor “injection” was significant for four genes upregulated by the bacterin injection: *ccl13*, *cish, znrf1,* and *bcl3* (Figures 4A–D). There was no significant interaction between diet and injection across the investigated genes. The mRNA levels of *interferon regulatory factor 1* (*irf1*) and *NF-kappa-B inhibitor alpha* (*nfkbia*) were not different among groups (Figures 3E, F).

The diet-matched bacterin vs. PBS control FC results are shown in Supplementary Figure S1; *tlr5* FC was significantly higher in the high-18:2ω6 group than in the switched-diet and high-18:3ω3 groups. *Clec3a* FC was significantly higher in the high-18:3ω3 group than in the switched-diet group, while *cish* FC was higher in the high-18:2ω6 than in the switched-diet group (Supplementary Figure S1). The bacterin vs. PBS control FC values of all the remaining targeted transcripts were not significantly different between diet groups.

### 3.3 Head kidney fatty acid profiles

At week 8 (see Figure 1 for experimental design), the head kidney FA profiles of salmon fed with high-18:3ω3 and high-18:2ω6 diets reflected the dietary composition (Table 3). Those fed the switched-diet showed some FA similarity to those fed the high-18:3ω3 diet (e.g., 18:2ω6 and 20:4ω3); however, they were intermediate between high-18:3ω3 and high-18:2ω6 groups for some FAs such as 18:3ω3 (Table 3). Both 18:2ω6 and DGLA (20:3ω6) were significantly higher in the high-18:2ω6 than in the other groups. The levels of ARA were not significantly different across the groups. The levels of 18:3ω3 were significantly higher in high-18:3ω3 than in the other groups, intermediate in the switched-diet group, and lowest in the high-18:2ω6 fed fish. The levels of EPA and DHA were not different across dietary treatments (Table 3).

**TABLE 3:** Fatty acid profiles (% total FAs^a^) of the head kidney tissues of Atlantic salmon after 8 weeks of the experimental diet regimens.

FA composition (% total FAs)	High-18:3ω3	Switched-diet	High-18:2ω6	*p*-value
14:0	1.44 ± 0.08	1.43 ± 0.13	1.41 ± 0.09	0.975
16:0	14.27 ± 0.43	15.5 ± 1.37	13.8 ± 0.31	0.360
18:0	4.58 ± 0.14	5.1 ± 0.44	4.81 ± 0.14	0.437
18:2ω6	13.2 ± 0.42^b^	13.75 ± 0.81^b^	20.42 ± 0.69^a^	*p* < 0.001
20:2ω6	0.86 ± 0.04^b^	0.84 ± 0.06^b^	1.28 ± 0.07^a^	*p* < 0.001
20:3ω6 (DGLA)	0.96 ± 0.05^b^	1.16 ± 0.1^b^	2.14 ± 0.21^a^	*p* < 0.001
20:4ω6 (ARA)	1.45 ± 0.14	2.01 ± 0.29	2.41 ± 0.35	0.065
18:3ω3	9.6 ± 0.58^a^	6.39 ± 0.56^b^	2.19 ± 0.1^c^	*p* < 0.001
18:4ω3	1.66 ± 0.09^a^	1.35 ± 0.16^a^	0.82 ± 0.05^b^	*p* < 0.001
20:4ω3	1.3 ± 0.07^a^	1.11 ± 0.1^a^	0.66 ± 0.1^b^	*p* < 0.001
20:5ω3 (EPA)	3.01 ± 0.28	3.38 ± 0.39	2.62 ± 0.26	0.252
22:6ω3 (DHA)	8.36 ± 0.85	10.06 ± 1.45	10.28 ± 1.14	0.460
ΣSFA^b^	21.27 ± 0.65	23.12 ± 1.8	21.11 ± 0.36	0.385
ΣMUFA^c^	35.08 ± 1.2	33.57 ± 1.93	32.74 ± 1.63	0.593
ΣPUFA^d^	43.49 ± 1.34	43.2 ± 3.3	46.03 ± 1.38	0.614
P/S	2.06 ± 0.09	1.98 ± 0.19	2.18 ± 0.05	0.551
Σω3	25.67 ± 1.23^a^	23.93 ± 2.48^a^ ^,^ ^b^	17.7 ± 1.32^b^	*p* < 0.001
DHA/EPA	2.81 ± 0.15^b^	2.93 ± 0.16^b^	3.9 ± 0.21^a^	*p* < 0.001
Σω6	17.17 ± 0.36^b^	18.56 ± 1.01^b^	27.58 ± 0.46^a^	*p* < 0.001
ω6/ω3	0.68 ± 0.03^b^	0.88 ± 0.13^b^	1.62 ± 0.11^a^	*p* < 0.001
EPA/ARA	2.09 ± 0.08^a^	1.79 ± 0.2^a^	1.16 ± 0.08^b^	*p* < 0.001
DHA/ARA	5.79 ± 0.22^a^	5.05 ± 0.39^a^ ^,^ ^b^	4.42 ± 0.17^b^	*p* < 0.001
DGLA/ARA	0.68 ± 0.03^b^	0.65 ± 0.07^a^ ^,^ ^b^	0.95 ± 0.08^a^	*p* < 0.001
EPA + DGLA/ARA	3.69 ± 0.25	4.03 ± 0.34	3.57 ± 0.22	0.489

a Mean ± s.e. (n = 9).

b Sum saturated fatty acids.

c Sum monounsaturated fatty acids.

d Sum polyunsaturated fatty acids.

At week 8, ΣSFA, ΣMUFA, and ΣPUFA were not different across dietary treatments, while Σω3 was significantly higher in high-18:3ω3 than in high-18:2ω6 fed fish and intermediate (and not significantly different from the other diet groups) in the switched-diet group (Table 3). Σω6 and ω6/ω3 ratios were significantly higher in high-18:2ω6 than those in the other groups, while EPA/ARA and DHA/ARA ratios were significantly higher in high-18:3ω3 than those in high-18:2ω6 (Table 3). DGLA/ARA was significantly higher in high-18:2ω6 than in high-18:3ω3 but showed no significant difference between the switched-diet and the other diet groups. EPA + DGLA/ARA was not significantly different among the dietary treatments (Table 3).

By comparing week 4 (fed the high-18:2ω6) with week 8 FA profiles of the switched-diet group (Supplementary Table S2), both 18:2ω6 and 20:3ω6 significantly decreased after the second 4 weeks (i.e., feeding the high-18:3-ω3 diet; week 8). Also, 18:3ω3 significantly increased in week 8 compared with week 4 in the switched-diet group. EPA, DHA, and ARA were not significantly different between the two sampling time points (i.e., week 4 vs. week 8 for the switched-diet group; Supplementary Table S2). Σω6 was significantly higher at week 4 than at week 8 in the switched-diet group (Supplementary Table S2).

### 3.4 Multivariate and correlation analyses

**FIGURE 5:**
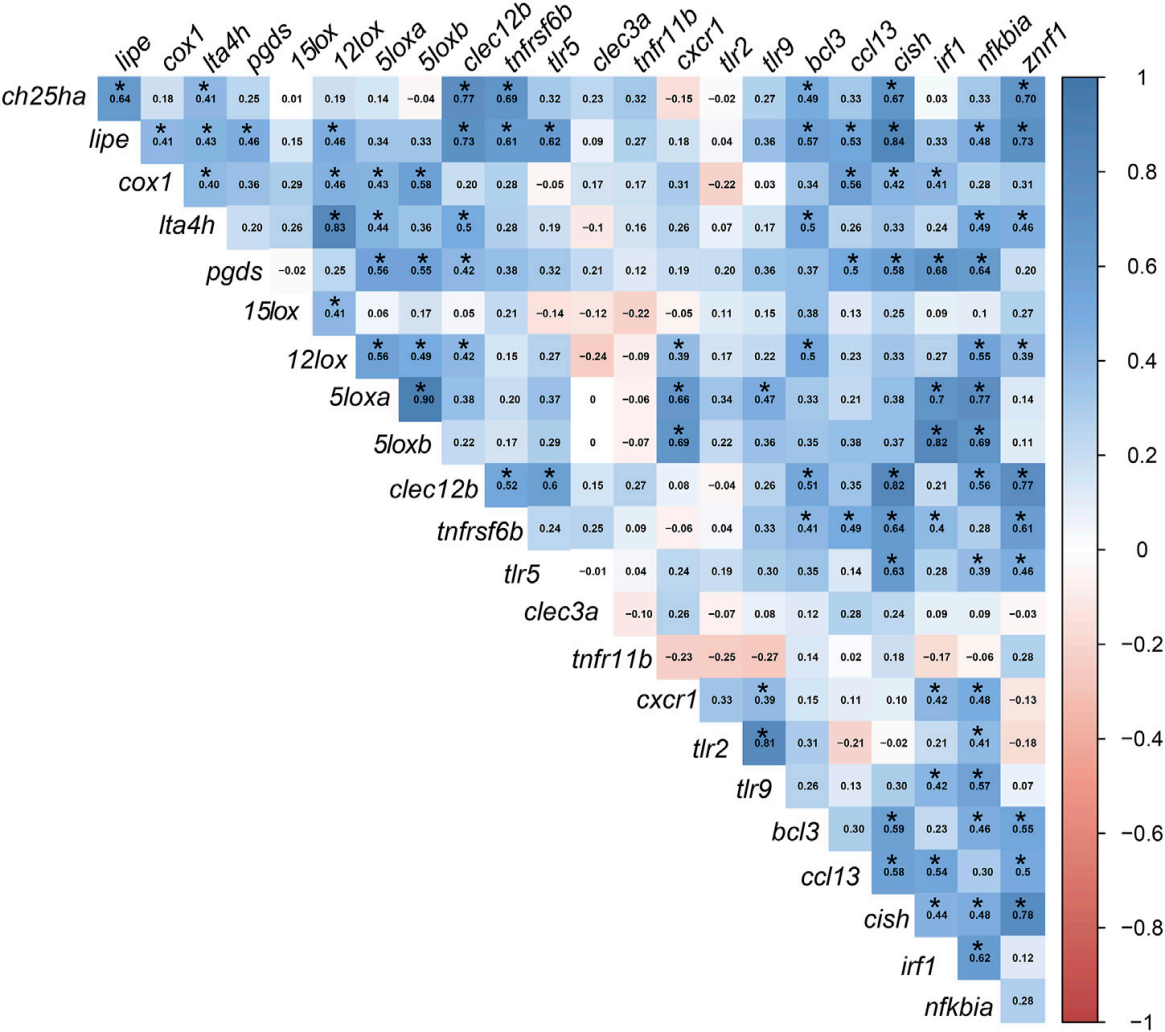
Pearson’s correlation coefficient (r) matrix for the fold-change of the targeted transcripts. FCs were calculated using individual RQ (n = 9) of the bacterin-stimulated individual/average RQs of the PBS-injected fish for each group. Created using the “corrplot” R-software package. Significant correlations with *p* ≤0.05 are indicated by an asterisk (*) on the r values in the cells (blue for positive correlations and red for negative correlations).

The FC [FC: individual RQ of the bacterin-stimulated fish/average RQs of the PBS-injected fish for each diet group] transcript-to-transcript correlation is shown in Figure 5 with significance, *p*-value < 0.05, indicated with asterisks. The FCs of several transcripts classified as biomarkers involved in lipid metabolism and inflammation response were correlated positively together. For example, *ch25ha* was correlated to both *lipe* and *lta4h*. Other examples are the *lipe* positive correlation to *cox1*, *lta4h*, *pgds*, and *12lox*; the *cox1* positive correlation to *lta4h*, *12lox*, *5loxa*, and *5loxb.* Furthermore, the following lipid and inflammation-relevant gene expression correlations were observed: *pgds* positively correlated with *5loxa* and *5loxb*; *12lox* positively correlated with *15lox*, *5loxa*, and *5loxb*; *5loxa* and *5loxb* positively correlated with one another (Figure 5).

Significant correlations between lipid metabolism/inflammation-relevant genes and immune-relevant genes were also observed. For example, *ch25ha* and *lipe* FCs were positively correlated with FCs of immune receptor-relevant transcripts (i.e., *clec12b* and *tnfrsf6b*) and immune effector-relevant transcripts (i.e., *bcl3* and *znrf1)*. Also, *cox1* (lipid metabolism and inflammation) correlated positively with immune regulator-relevant biomarkers (i.e., *ccl13*, *cish*, and *irf1*). The FC levels of *lta4h* and *pgds* were correlated positively with *clec12b* and *nfkbia*. As well, both *5loxa* and *5loxb* were positively correlated with *cxcr1*, *irf1*, and *nfkbia* (Figure 5).

Several transcripts encoding proteins classified as immune receptors were correlated to immune regulator-relevant transcripts (e.g., FCs of *clec12b*, *tnfrsf6b*, and *tlr5* correlated positively with *cish* and *znrf1*; *tlr5*, *cxcr1*, *tlr2*, and *tlr9* correlated positively with *nfkbia*). Additional immunoregulator transcripts were correlated positively to lipid metabolism and immune receptor biomarkers, for example, the FCs of *znrf1* were correlated with *ch25ha*, *lipe*, *lta4h*, *12lox*, *clec12b*, *tnfrsf6b*, *tlr5*, *bcl3*, *ccl13*, and *cish*. Also, *irf1* correlated positively with *cox1*, *pgds*, *5loxa* and *5loxb*, *tnfrsf6b*, *tlr5*, *bcl3*, and *ccl13*.

**FIGURE 6:**
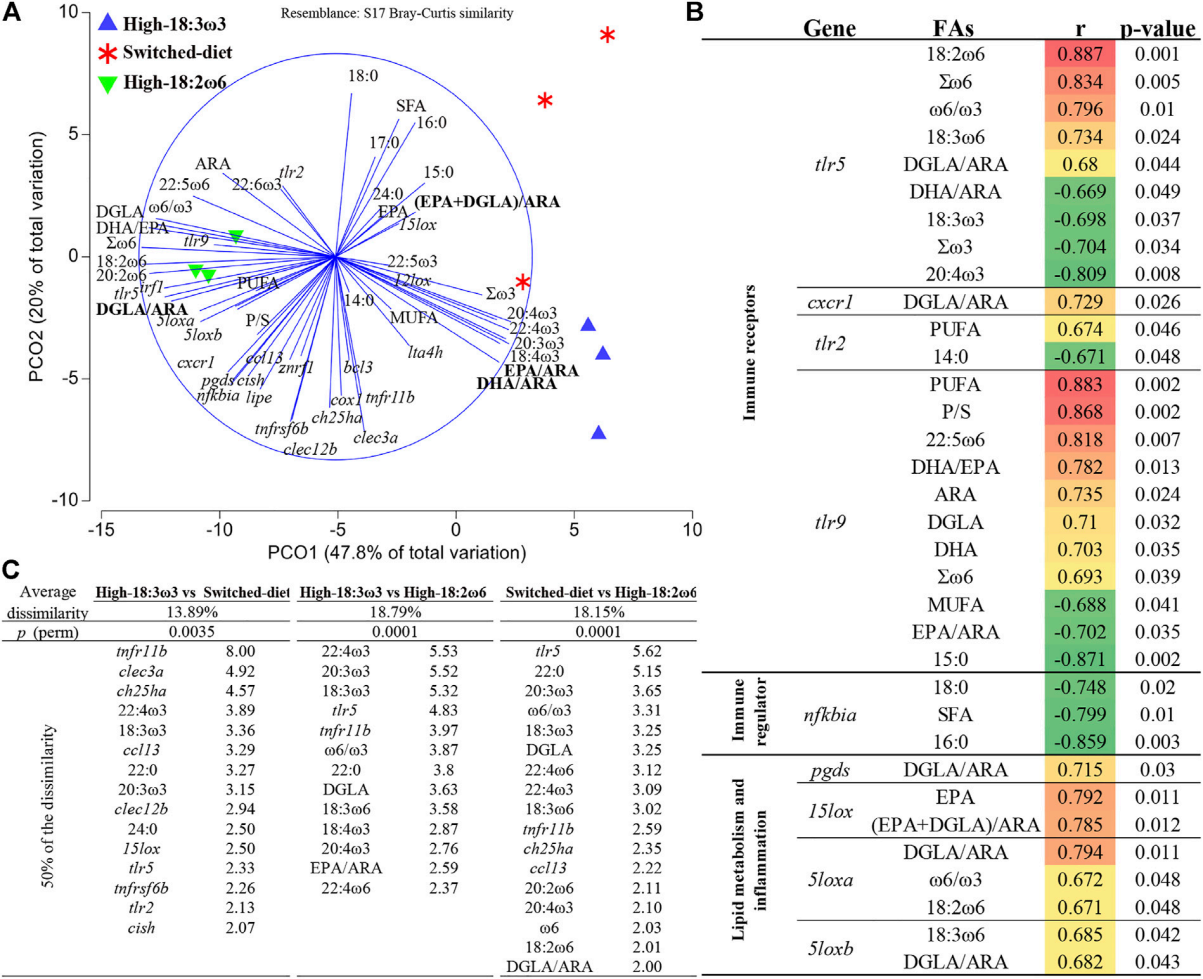
**(A)** Principal coordinates analysis (PCoA) for head kidney FAs and fold-change (FC) of each targeted transcript using tank mean (tank numbers = 3 for nine individual fish). **(B)** Significant correlation between tank means of gene expression FCs and FAs. **(C)** *p* (perm) is the statistical significance value of the pairwise comparison using permutational multivariate ANOVA (PERMANOVA), and a list of contributing variables (top 50%) that were obtained through similarity of percentage analysis (SIMPER).

As shown in Figure 6A, the PCoA revealed a clear separation between the high-18:2ω6 group (negatively loaded on PCO1) and the high-18:3ω3 and switched-diet groups (positively loaded on PCO1). PCO1 explained 47.8% of the variation, while PCO2 explained 20.0% of the variation. The vectors representing FAs showed a dietary reflection for both high-18:2ω6 and high-18:3ω3 groups. The switched-diet and high-18:3ω3 individuals were plotted closely together on PCO1, but these groups were separated on PCO2 (Figure 6A). Notably, the switched-diet individuals plotted closer to the (EPA + DGLA)/ARA ratio. The DGLA/ARA vector was plotted closer to the high-18:2ω6 group. The vectors DHA/ARA and EPA/ARA were associated with the high-18:3ω3 group. The FC vectors of *15lox*, *12lox*, and *lta4h* were loaded closer to the switched-diet and high-18:3ω3 individuals. The FC vectors of *tlr2* and *tlr9*, *irf1*, *5loxa* and *5loxb*, *cxcr1, pgds*, and *nfkbia* loaded closer to the high-18:2ω6 individuals, while the vector representing *15lox* FC loaded closer to the switched-diet individuals and the (EPA + DGLA)/ARA vector.

Figure 6B shows all significant (*p*-value <0.05) correlations between FAs and expression of immune/inflammation and lipid metabolism-relevant transcripts. For example, DGLA/ARA was positively correlated with *pgds*, *5loxa*, *5loxb*, *tlr5*, and *cxcr1* FCs. As another example, the ω6/ω3 ratio was positively correlated with *5loxa* and *tlr5*. Both EPA and (EPA + DGLA)/ARA were correlated positively with *15lox* FC (Figure 6B). The FCs of *5loxa* and *5loxb* were positively correlated with 18:2ω6 and 18:3ω6, respectively. Furthermore, the FC of transcripts encoding two pattern recognition receptors (PRR; i.e., *tlr5* and *tlr9*) had the highest number of significant correlations to FAs, and they were correlated positively with Σω6. Also, *tlr5* FCs were positively correlated with ω6/ω3 and two ω6-FAs (i.e., 18:2ω6 and 18:3ω6) and negatively correlated with Σω3, DHA/ARA, and two ω3-FAs (i.e., 18:3ω3 and 20:4ω3). FC values of *tlr9* were positively correlated with PUFA, polyunsaturated/saturated FAs (P/S), 22:5ω6, DHA/EPA, ARA, DGLA, DHA, and Σω6. Also, *tlr9* was negatively correlated with MUFA, EPA/ARA, and 15:0. FCs of *tlr2*, another PRR, were positively correlated with PUFA and negatively correlated with 14:0. The FCs of *nfkbia* were negatively correlated with 18:0, SFA, and 16:0 (Figure 6B).

In Figure 6C, the pairwise comparison using PERMANOVA showed highly significant differences between all groups. The similarity of percentage analysis (SIMPER) showed that the highest average dissimilarity (18.8%) was observed between high-18:3ω3 and high-18:2ω6; top contributors to this dissimilarity were 22:4ω3, 20:3ω3, 18:3ω3, *tlr5*, and *tnfr11b* (percent of contribution listed next to each variable in Figure 6C)*.* The average dissimilarity between switched-diet and high-18:2ω6 was 18.2%, and the top contributors to this dissimilarity were *tlr5*, 22:0, 20:3ω3, ω6/ω3, DGLA, and 18:3ω3. The lowest average dissimilarity (i.e., 13.9%) was observed between high-18:3ω3 and switched-diet groups; the top contributors to this dissimilarity were *tnfr11b*, *clec3a*, *ch25ha*, 22:4ω3, and 18:3ω3 (Figure 6C). Across the three comparisons, *tnfr11b*, *tlr5,* 22:4ω3*,* 20:3ω3, and 18:3ω3 were within the top contributors to dissimilarities (Figure 6C).

In Supplementary Figure S2A, the PCoA of the RQs showed some overlap between the PBS-injected groups across different dietary treatments and the bacterin-injected group fed the switched-diet regimen. The PERMANOVA results showed a highly significant (*p*-value <0.01) difference between the bacterin-injected groups and the PBS-injected group for the high-18:3ω3 and high-18:2ω6 groups but not for the switched-diet group (*p*-value = 0.065) (Supplementary Figure S2B). Both *ccl13* and *ch25ha* were within the top three contributors to the dissimilarities between the bacterin and PBS-injected groups in the high-18:2ω6 and high-18:3ω3 dietary treatments. In Supplementary Figure S2C, the PERMANOVA results showed that the bacterin-injected groups were not significantly different from each other.

The PCoA biplot (Supplementary Figures S2A–S2B) of head kidney FA proportions and ratios reflected the diets fed, with switched-diet individuals plotting closer to the high-18:3ω3 individuals. The latter was associated with the DHA/ARA vector, while the former was associated with the EPA + DGLA/ARA vector. High-18:3ω6 individuals were associated with ω6 FA proportions and the DGLA/ARA vector.

## 4 Discussion

### 4.1 Head kidney gene expression

#### 4.1.1 Lipid metabolism and inflammation response

In the current study, some lipid metabolism-related genes (e.g., *lipe* and *ch25ha*) were significantly upregulated in response to bacterin in both high-18:2ω6 and high-18:3ω3 groups, with no significant response in the switched-diet group (Figures 2A,B). Some inflammatory eicosanoid-relevant biomarkers (i.e., *5loxb* and *5loxa*) were significantly downregulated in response to *R. salmoninarum* bacterin (Figures 2C,D). Lipids, besides having roles in membranes, also serve as a source of energy and as signaling molecules during infection and inflammation (Teng et al., 2017). Furthermore, host lipid metabolism manipulation is part of the infection mechanisms of some pathogens (Helms, 2006; Wenk, 2006; Wymann and Schneiter, 2008; Zhou et al., 2021). Therefore, the regulation of lipid metabolism might affect susceptibility to disease and the outcome of infections. Infection and inflammation lead to an array of physiological changes known as the acute phase response, resulting in the modulation of lipid and lipoprotein metabolism (Jahangiri, 2010). Lipid metabolism changes such as increased levels of total cholesterol, free cholesterol, and phospholipid were observed in the inflammatory responses of mice to wounds, lipopolysaccharides (LPS), and killed *Bordetella pertussis*, suggesting serum lipids as a sensitive index for the acute inflammatory phase (Kitagawa et al., 1992). Furthermore, changes in dietary lipids modulated the response of different teleost fish [e.g., Atlantic salmon, common carp (*Cyprinus carpio*)] to viral and bacterial challenges, respectively (Martinez-Rubio et al., 2012; Nguyen et al., 2021). Also, lipid metabolism is regulated differently in M1 and M2 macrophages to promote their primary functions: pro-inflammatory and anti-inflammatory, respectively (Batista-Gonzalez et al., 2020). Collectively, these studies suggest that the dietary FA profile influences the host immune response. The current study’s results help to elucidate the role of lipid metabolism in the regulation of immune response in immune-challenged Atlantic salmon by the integrative analysis and interpretation of gene expression and FA composition data.

Both *ch25ha* and *lipe* were significantly upregulated in the bacterin immune-stimulated high-18:2ω6 and high-18:3ω3 groups but not the switched-diet group (Figures 2A, B). Furthermore, *ch25ha* was one of the top contributors to the dissimilarities between the switched-diet group and both the high-18:2ω6 and high-18:3ω3 groups (Figure 6C and Supplementary Figure S2C). CH25H is a membrane-associated enzyme that catalyzes the formation of 25-hydroxycholesterol from cholesterol (Xu et al., 2020). Furthermore, CH25H showed an interferon-independent antiviral and immunoregulatory activity in teleosts [e.g., zebrafish and Chinese tongue sole (*Cynoglossus semilaevis*)] (Pereiro et al., 2017; Xu et al., 2020). Also, *ch25ha* was significantly upregulated by *Piscirickettsia salmonis* infection in the head kidneys of Atlantic salmon parr (Xue et al., 2021). CH25H is suggested as an inflammatory signaling amplifier (Gold et al., 2014) and was found to be induced by dietary ARA (pro-inflammatory FA) in the brains of Chinese tongue sole (Xu et al., 2020). Also, *ch25ha* was previously microarray-identified and qPCR-confirmed as induced in the head kidneys of Atlantic salmon responses to formalin-killed (i.e., 5×10^7^ cells/kg; similar to the current study) and live *R. salmoninarum* (Eslamloo et al., 2020b; 2020a). Another lipid metabolism-relevant enzyme, LIPE, is a rate-limiting enzyme for mobilizing adipose tissue-derived free FAs and can target intracellular triacylglycerol to ensure consistent energy supply (Watt and Steinberg, 2008). Macrophages use free FAs released from intracellular triacylglycerol as energy substrates, mediators in cell signaling, and in membrane synthesis (Ghosh, 2011; Radovic et al., 2012). Furthermore, mammalian macrophage peroxisome proliferator-activated receptor (PPAR)-beta could be activated by low-density lipoprotein, and it was hypothesized that the absence of triglyceride lipase in macrophages could limit PPAR signaling (Lee et al., 2006; Radovic et al., 2012). In mouse macrophages, lipase deficiency attenuated the release of pro-inflammatory cytokines (i.e., chemokine ligand 1 and IL-6) and increased the secretion of the anti-inflammatory IL-10 (Aflaki et al., 2011; Lammers et al., 2011; Radovic et al., 2012). Also, *lipe* was microarray-identified and qPCR-confirmed as induced in response to *R. salmoninarum* bacterin in the head kidneys of Atlantic salmon (Eslamloo et al., 2020b). While the immune-related functions of *ch25ha* and *lipe* have yet to be fully characterized in Atlantic salmon, the bacterin-triggered upregulation of *ch25ha* and *lipe* in the high-18:2ω6 and high-18:3ω3 groups in the current study further confirms the involvement of these transcripts in the immune response of Atlantic salmon to *R. salmoninarum*. The attenuated responses of *ch25ha* and *lipe* in the switched-diet group may suggest suppressive effects on the inflammatory response to the bacterin. It is worth mentioning that dietary switching in mice, from obesogenic to normal diet (vs. continued obesogenic diet), decreased systemic blood concentration of pro-inflammatory cytokines (e.g., IL-1a) and increased that of anti-inflammatory cytokines (e.g., IL-4) (Sun et al., 2021). The impact of abrupt intake differences in dietary FAs (i.e., dietary switching) on teleost fish immune/inflammatory response warrants further investigation.

Although *5loxb* and *5loxa* were significantly downregulated overall by bacterin injection (Figures 2C,D), the response was muted for high-18:2ω6 fish. 5LOX is responsible for the biosynthesis of LTA4, that is, an inducing factor for pro-inflammatory cytokines (Fiorucci et al., 2001). The PCoA showed that *5loxa* and *5loxb* vectors were loaded closer to the high-18:2ω6 individuals (Figure 6A). This is further supported by the significant positive correlation of both *5loxa* and *5loxb* with DGLA/ARA (Figure 6B). Furthermore, *5loxa* was negatively correlated with EPA/ARA in the liver of Atlantic salmon (Caballero-Solares et al., 2020). As well, *5loxa* and *5loxb* were correlated positively with EPA and DHA in the head kidney of Atlantic salmon fed with different EPA and DHA levels alongside either high-ω6 or high-ω3 C_18_ FAs (Katan et al., 2020). Atlantic salmon fed with microbial oil upregulated *5loxa* in the muscle tissue when compared with those fed with FO (Osmond et al., 2021). Altogether, the current study results further suggest the involvement of LOX in regulating the inflammatory process, as previously suggested in the study of Dennis and Norris (2015), with or without direct immune system stimulators. This avenue of study could be explored in the future to develop therapeutic strategies for modulating the inflammatory response during infection (e.g., *R. salmoninarum*). *5loxa*, *5loxb*, *12lox,* and *15lox* were not previously identified as responsive to *R. salmoninarum* (Eslamloo et al., 2020b; 2020a); rather, these qPCR assays were developed for the current study to explore the interaction of dietary FAs with eicosanoid pathways during the bacterin challenge. Although *cox1* (investigated in the current study) did not show a significant response to the bacterin across the diets, the FC was significantly correlated with *5loxa* and *5loxb*. Also, *5loxa* and *5loxb* were positively correlated to well-known inflammatory and immune-relevant biomarkers (i.e., *cxcr1*, *irf1*, and *nfkbia*) (Bonelli et al., 2019; Mattos et al., 2020; Tan et al., 2020) (Figure 5). The inter-relationship between cyclooxygenase and lipoxygenase pathways requires further study in teleosts, notably for developing anti-inflammatory therapeutics and diets, especially with the observed higher magnitude of downregulation in both high-18:3ω3 and switched-diet fed groups.

### 4.1.2 Immune receptors

The transcript levels of *tnfrsf6b* were significantly induced (∼1.9–2.7-fold) by the bacterin in all dietary treatments (Figure 3A), however, with higher FC and significance levels in the high-18:2ω6 and high-18:3ω3 groups than in the switched-diet group. Also, *tnfrsf6b* was one of the top biomarkers contributing to the dissimilarities between the bacterin and PBS-injected groups with different dietary treatments (Supplementary Figure S2B). TNFRSF6B was suggested as a biomarker for pathogen-derived inflammation, as it plays an important role in sepsis pathogenicity in humans (Kim et al., 2012). Also, *tnfrsf6b* was found upregulated for both viral and bacterial pathogen-associated molecular patterns (PAMPs) in Atlantic salmon mononuclear phagocytes (Iliev et al., 2019). Similarly, in the Atlantic salmon head kidney, *tnfrsf6b* was induced in response to live and formalin-killed *R. salmoninarum* (Eslamloo et al., 2020b; 2020a). The findings of our current and previous studies (Eslamloo et al., 2020b; 2020a) indicate the importance of this receptor for the responses of Atlantic salmon to *R. salmoninarum* or its derived antigens and suggest it as a potential candidate for developing molecular diagnostic tools specific for pathogen-associated inflammation [as previously suggested in the study by Kim et al. (2012)].

The transcript levels of *clec12b* were significantly upregulated by the bacterin injection in the high-18:3ω3 and high-18:2ω6 groups (compared with diet-matched PBS controls) but not in the switched-diet group (Figure 3B). Furthermore, *clec12b* was one of the key genes contributing to the dissimilarity between high-18:3ω3 and switched-diet groups (Figure 6C). *Clec12b* was found upregulated in the head kidney of Atlantic salmon with *R*. *salmoninarum* bacterin challenge (Eslamloo et al., 2020b)*.* Although still poorly understood, mammalian CLEC12B has been proposed as an inhibitory receptor with roles in preventing immune cell hyperactivation through its possible interaction with caveolin-1 (CAV1) (Hoffmann et al., 2007; Tone et al., 2019). CAV1 forms the subdomain of lipid rafts (Lajoie and Nabi, 2010), and lipid rafts are membrane microdomains involved in the formation and amplification of cell signaling and coalescence of receptors (Gagliardi et al., 2021). It was previously suggested that the disruption of membrane raft formation blocks infection and intracellular entry of virulence factors (Gagliardi et al., 2021). Assuming this Atlantic salmon CLEC12B has a similar function as its mammalian putative ortholog, the lack of response of *clec12b* to the bacterin in the switched-diet salmon may suggest a lesser need for compensatory immunosuppressive mechanisms; however, further research is required to investigate the potential involvement of lipid rafts in the observed attenuated responses in the switched-diet group. Another member of the C-type lectin family studied herein*, clec3a*, was downregulated by the bacterin only in the switched-diet group (Figure 3D), and *clec3a* FC was significantly lower in the switched-diet group than in the high-18:3ω3. Furthermore, *clec3a* was one of the top genes contributing to the dissimilarity between fish injected with bacterin and PBS in the switched-diet group (Supplementary Figure S2B) and the second most influential gene contributing to the dissimilarity between high-18:3ω3 and switched-diet groups (Figure 6C). Mammalian CLEC3A has been reported to have antimicrobial activity (Elezagic et al., 2019). In our previous study, *clec3a* levels were suppressed in the high dose (5 × 10^7^ cells kg^−1^; that is, the same dose used in the current study) *R*. *salmoninarum* bacterin-injected group than in the pre-injection group (Eslamloo et al., 2020b) but not when compared with the PBS-injected controls. The observed downregulation of *clec3a* might be part of the switched-diet-associated immunomodulation response to the bacterin. The regulation of *clec12b* and *clec3a* anti-bacterin responses (i.e., suppression of *clec12b* induction in the switched-diet group and downregulation of *clec3a* only in the switched-diet group) supports their involvement in regulating immune responses (Hoffmann et al., 2007; Tone et al., 2019). Also, these genes may be useful molecular biomarkers in future studies of dietary influence on antibacterial responses (e.g., the development of diets that manipulate antibacterial resistance and the associated pro-inflammatory responses).

The downregulation of *cxcr1* upon *R. salmoninarum* bacterin challenge was in alignment with our previous findings (Eslamloo et al., 2020b; 2020a), and it was observed across all the dietary treatments (Figure 3F). Also, this transcript showed a close association with high-18:2ω6 individuals based on our PCoA and was positively correlated with DGLA/ARA (Figures 6A,B). Furthermore, it was significantly and positively correlated with *12lox*, *5loxa*, and *5loxb* (Figure 5). CXCR1 is a chemokine receptor involved in neutrophil recruitment through activation of chemotaxis signaling cascades (Ha et al., 2017). In mammals, CXCR3 of T-lymphocytes was upregulated with a saturated FA-enriched diet (Mauro et al., 2017; Radzikowska et al., 2019). The consistency of *cxcr1* response across studies (Eslamloo et al., 2020b; 2020a) and diets to the bacterin (in the current study) suggests neutrophil recruitment may be suppressed in bacterin-exposed fish and might shed light on the immune-suppressive properties of *R. salmoninarum* (previously described in the study of Grayson et al. (2002)). Also, it may suggest *cxcr1* as a candidate molecular biomarker in evaluating vaccination strategies for BKD. The CXCR1 interaction with FAs (based on the reported correlation with DGLA/ARA and eicosanoid-relevant biomarkers, for example, *12lox* and *5loxa*) requires further investigation in teleost fish, especially with higher dietary ω6 FAs.

Atlantic salmon *tlr5* was significantly induced by the bacterin in both the high-18:2ω6 and high-18:3ω3 groups (Figure 3); nevertheless, *tlr5* FC was significantly higher in the high-18:2ω6 group than both other groups (Supplementary Figure S1). Mammalian TLR5 was found to have several innate immune response-related functions, including stimulation of the production of pro-inflammatory cytokines, in addition to its well-known role in the recognition of flagellated bacteria (Tallant et al., 2004; Feuillet et al., 2006; Porte et al., 2015; Yang and Yan, 2017). In addition to the membrane TLR5, soluble TLR5 has been identified in different teleost species (Tsoi et al., 2006; Tsujita et al., 2006; Wangkahart et al., 2019). Soluble TLR5 (encoded by the transcript targeted in the current study based on Tsoi et al. (2006)) was suggested as an acute-phase protein with a flagellin-recognition activity that could amplify NF_K_B activation in rainbow trout (Tsukada et al., 2005). *R. salmoninarum* is an intracellular, non-motile bacterium (Delghandi et al., 2020); thus, *R. salmoninarum* bacterin-dependent induction of Atlantic salmon *tlr5* seen herein may be associated with its function beyond pathogen recognition [e.g., inflammatory signaling amplification as suggested in Tsujita et al. (2006) and Chamberlain et al. (2012)]. Our results indicate the experimental dietary treatments significantly influenced the *tlr5* response to the *R. salmoninarum* bacterin. First, *tlr5* mRNA levels were higher in the bacterin-stimulated high-18:2ω6 salmon than in the bacterin-stimulated high-18:3ω3 salmon (Supplementary Table S1). Concurrently, the FC induction of *tlr5* by the bacterin was significantly higher in the fish fed with the high-18:2ω6 diet (∼6.7-FC) than in those fed with the high-18:3ω3 diet (∼2.5-FC) or switched-diet (∼2.2-FC) (Supplementary Figure S1). In the multivariate analyses (Figure 6A), *tlr5* was found to be one of the top contributors to the dissimilarity between the switched-diet and high-18:2ω6 groups and between the high-18:3ω3 and high-18:2ω6 groups (Figure 6C). The *tlr5* vector was plotted near the high-18:2ω6 salmon and loaded close to the ARA vector. ARA (one of the C_18_ ω6 desaturation and elongation products) is a pro-inflammatory FA with roles in modulating the function of several receptors, ion channels, and enzymes (Tallima and El Ridi, 2017). Furthermore, *tlr5* was positively correlated with some ω6 FAs (e.g., 18:2ω6) and DGLA/ARA, and it was negatively correlated with Σω3 FAs (Figure 6B). Our findings suggest that *tlr5* induction upon *R. salmoninarum* bacterin stimulation is strengthened under a dietary and tissue FA profile typically regarded as pro-inflammatory (i.e., high ω6 diet and high ARA levels in the tissue). Interestingly, *tlr9* did not respond significantly to the *R. salmoninarum* bacterin but showed a positive correlation with ARA and Σω6 and a negative correlation with DHA/ARA and EPA/ARA (Figure 6B). There is growing evidence supporting the influence of lipid contents on membrane receptor functions (Sunshine and Iruela-Arispe, 2017), and it was previously reported that FAs may activate (e.g., saturated FAs) or inhibit (ω3-FAs) Toll-like receptor signaling (Fessler et al., 2009). It is noteworthy that the anti-inflammatory effects of ω3 FAs may be mediated through the activation of PPAR-gamma, interfering with TLR4-associated NF_K_B signaling (Calder, 2013). The PRR–FA interaction was previously reported in a murine cell model (i.e., RAW 264.7; a monocyte/macrophage-like cell line) (Lee et al., 2001). However, PRR-FA interactions in teleost species are not well understood.

#### 4.1.3 Immune effectors and transcription factors

In the present study, *ccl13* showed consistent upregulation (Figure 4A) by the bacterin stimulation, regardless of the diet [∼5.9 FC for the switched diet (with the lowest level of significance), ∼8.4 FC for the high-18:2ω6, and ∼8.6 FC for the high-18:3ω3]. Also, it was consistently one of the top contributors to dissimilarities between the bacterin and PBS-injected groups across all diets (Supplementary Figure S2B). *Ccl13* was previously found to be upregulated in the head kidney of Atlantic salmon following *R. salmoninarum* bacterin challenge and with live *R. salmoninarum* infection (Eslamloo et al., 2020b; 2020a)*.* Mammalian CCL13, as well, showed antibacterial activity against *Pseudomonas aeruginosa* (Cossio-Ayala et al., 2017). In both Atlantic salmon and Atlantic cod macrophages, *ccl13* was found to be induced by polyinosinic: polycytidylic acid [poly(I:C)] stimulation (Eslamloo et al., 2016, 2017). CCL13 acts as a ligand to CCR2, suggesting its role in dendritic cell (DC) maturation and function (Chiu et al., 2004; Mendez-Enriquez and García-Zepeda, 2013). The consistent response of *ccl13* to the bacterin across diets further suggests CCL13 as a possible target for anti-inflammatory therapeutics (Mendez-Enriquez and García-Zepeda, 2013) and in evaluating vaccination efficacy, regardless of the dietary treatment.

*Cish* showed significant upregulation with the bacterin only in the high-18:3ω3 and high-18:2ω6 groups (Figure 4B), with significant FC induction in the high-18:2ω6 when compared to switched-diet (Supplementary Figure S1). *Cish* was found to be upregulated in response to a *R. salmoninarum* bacterin challenge in the head kidney of Atlantic salmon (Eslamloo et al., 2020b). CISH belongs to a suppressor of cytokine signaling family, as it negatively regulates further cytokine induction (Takeshima et al., 2019). Also, in humans, it plays a role in the regulation of inflammatory response through the IL2 signaling pathway and suppresses immune response by interaction with cytokine receptors (Yoshimura et al., 2018). Furthermore, human CISH seems to be critical for T-cell proliferation and survival during the response to infection (e.g., tuberculosis and severe malaria) (Leonard, 2001; Khor et al., 2010). *Cish* was found upregulated in the head kidney of rainbow trout challenged with *Yersinia ruckeri* for 24 h (Maehr et al., 2014). The lack of upregulation of *cish* to the bacterin in the switched-diet group aligns with the *clec12b* expression profile and further supports the notion that the switched-diet treatment reduced the need for compensatory immunosuppressive mechanism to limit the immune response to the bacterin, based on an assumption that salmon CISH has a similar function to the mammalian CISH.

Membrane FAs and consequent lipid mediators may function as initiators of leukocyte recruitment, which attract the first leukocytes to an inflammation site (Sadik and Luster, 2012); thereafter, the first responding immune cells recruit other immune cells through chemoattraction (Sadik and Luster, 2012). Prostaglandins can be generated from ARA through the COX pathway (Sadik and Luster, 2012). In Atlantic salmon, *pgds* was found to be downregulated by *Piscirickettsia salmonis* infection in the head kidney (Xue et al., 2021), and lower levels of hepatic EPA/ARA were correlated with higher levels of *pgds* transcript in the liver (Caballero-Solares et al., 2020). The importance of eicosanoids and the chemoattractant cascades is highlighted herein by the significant positive correlation of *pgds* to cytokine-relevant biomarkers, *ccl13* and *cish*.

In the current study, *znrf1* showed significant upregulation with the bacterin only in the high-18:3ω3 and high-18:2ω6 groups (Figure 4C). Also, it was one of the top contributors to the dissimilarity between the bacterin-injected groups fed with high-18:3ω3 diet and switched-diet (Supplementary Figure S2C). *Znrf1* was found upregulated with *R. salmoninarum* bacterin stimulation (i.e., 5×10^7^ cells/kg) and live bacterium infection (Eslamloo et al., 2020b; 2020a). Mouse ZNRF1 interacts with CAV1 (the major constituent of caveolae/lipid rafts) and mediates its ubiquitination and degradation (Lee et al., 2017) in response to LPS. The ZNRF1–CAV1 axis enhances the production of pro-inflammatory cytokines (e.g., IL-6 and IL-1β) and inhibits the production of anti-inflammatory cytokines (e.g., IL-10) (Lee et al., 2017). Also, ZNRF1 deletion led to an increase in the resistance to endotoxic and microbial septic shock and an attenuated inflammatory response in mouse hematopoietic cells (Lee et al., 2017). Furthermore, it was previously reported that mouse ZNRF1 might regulate TLR4 activation and consequently the production of pro-inflammatory cytokines (Lee et al., 2017). The latter seems to be in agreement with the significant positive correlation between *znrf1* and *tlr5* in the present study (Figure 5). The unresponsiveness of *znrf1* in the switched-diet group supports the hypothesis that switching from a high 18:2ω6 diet to a high 18:3ω3 diet attenuated some of the Atlantic salmon’s innate immune response to the *R. salmoninarum* bacterin. However, the potential involvement of the lipid rafts (through CAV1) in the observed attenuated response in the switched-diet group requires further research. Our results revealed *clec12b*, *tlr5*, *znrf1*, and *cish* as suitable biomarkers for assessing dietary modulation of antibacterial immune responses in Atlantic salmon.

The bacterin-triggered upregulation of *bcl3* was only seen for the high-18:3ω3 group (Figure 4D). *Bcl3* was microarray-identified as upregulated with *R. salmoninarum* bacterin challenge in the head kidney of Atlantic salmon (Eslamloo et al., 2020b). In channel catfish gill, *bcl3* was found upregulated with columnaris disease (*Flavobacterium columnare*) and that was associated with suppression in NF_K_B signaling (Sun et al., 2012). Also, *bcl3* was found upregulated in the spleen of meagre (*Argyrosomus regius*) with LPS after 4 h post-stimulation (Monteiro et al., 2022). Overexpression of mammalian *bcl3* decreased NF_K_B activity, inflammatory response, and cell death of cortical tubule cells (Poveda et al., 2017). Mouse BCL3 mediates LPS tolerance and cytokine production susceptibility to the bacterial pathogen *Klebsiella pneumoniae* (Pène et al., 2011). Based on these collective results, we hypothesize that the observed induction of *bcl3* (only in the high-18:3ω3 group) may point to altered NF_K_B signaling, suggesting *bcl3* is associated with the ω3 FA-specific anti-inflammatory effect.

### 4.2 Head kidney fatty acid profile

EPA, DHA, and ARA were not different across the dietary treatments. However, the C_18_ FAs reflected the diet, especially in both the high-18:3ω3 and high-18:2ω6 groups. This suggests higher retention of longer chain FAs than the C_18_ FAs. In Atlantic salmon, muscle EPA + DHA was increasingly retained with a gradual decrease in dietary EPA + DHA (Bou et al., 2017). Also, muscle DHA and ARA (% of total FAs) of Atlantic salmon were not different between dietary treatments fed with graded levels of EPA + DHA (0.3% vs. 1%) and with different precursors (i.e., 18:2ω6 vs. 18:3ω3) (Emam et al., 2020). EPA plotted closer to the switched-diet and the high-18:3ω3 groups on PCO1 (explaining 47.8% of the variability; Figure 6A). This relates to the higher EPA in both the switched-diet and the high-18:3ω3 groups. DGLA (which is a precursor to an anti-inflammatory mediator (Santoli and Zurier, 1989; Gallagher et al., 2019)) was significantly higher in the high-18:2ω6 group than in the other groups, and it was associated with the high-18:2ω6 individuals. It was previously reported that salmon fed high 18:2ω6 diets elongated 18:2ω6 to higher amounts of DGLA when compared to ARA in muscle tissue (Emam et al., 2020). This result together with the current study further suggests the ability of salmon to balance FA profiles between inflammatory and less inflammatory FAs, even when only fed high 18:2ω6 diets.

By comparing the week 4 profile (fed high-18:2ω6) to week 8 (fed high-18:2ω6 and then high-18:3ω3) in the switched-diet group, salmon reflected the dietary C_18_ FAs (i.e., 18:2ω6 and 18:3ω3) in the head kidney FA profile in only 4 weeks. This highlights the role of dietary FA precursors in defining the FA profile of an immune-relevant organ (i.e., the head kidney). Interestingly, DGLA was significantly lower at week 8 than at week 4 in the switched-diet group, underlining the association of head kidney DGLA with a high ω6 diet. Concurrently, both EPA and DHA in the head kidney were not significantly different across groups, suggesting increased retention when fed lower levels (Bou et al., 2017; Emam et al., 2020).

### 4.3 Head kidney fatty acid ratios as indicators of inflammatory balance and their relevance to gene expression results

Since the fish head kidney is a main hematopoietic and lymphoid site, consisting of large populations of immune cells, its FA profiles may reflect these cells’ membrane composition, which influences various immune functions (e.g., antigen presentation and eicosanoid production) (Bell et al., 1996; Petrie-Hanson and Ainsworth, 2001; Yaqoob, 2003; Feng et al., 2009; Martinez-Rubio et al., 2013). Considering the activity of the derived eicosanoids (Allam-Ndoul et al., 2017; Gallagher et al., 2019), the DGLA/ARA, EPA/ARA, and (EPA + DGLA)/ARA ratios may be used as markers of the balance between anti-inflammatory (i.e., EPA and DGLA) and pro-inflammatory (i.e., ARA) FAs.

The vector representing DGLA/ARA was plotted closer to the high-18:2ω6 individuals in the multivariate space (Figure 6A). DGLA/ARA also showed a significant correlation with transcripts encoding eicosanoid-synthesizing enzymes (i.e., *pgds*, *5loxa*, and *5loxb*; Figure 6B), which further connects DGLA and ARA to the production of eicosanoids. DGLA (i.e., the ω6 anti-inflammatory FA in “DGLA/ARA”) attenuated the migration of chemokine-driven monocytes and the expression of cytokine-induced pro-atherogenic biomarkers in human macrophages (Gallagher et al., 2019); also, DGLA competes with ARA for COX and LOX enzymes (Wang et al., 2012). The observed significantly higher DGLA/ARA in the high-18:2ω6 group than in the high-18:3ω3 group (Table 3), together with the aforementioned correlations, suggest that individuals in the high-18:2ω6 group promoted elongation of 18:2ω6 to DGLA to prevent an excessive accumulation of pro-inflammatory ARA in the head kidney. This assumption might explain the observed comparable response to bacterin in most of the targeted biomarkers (e.g., *ch25ha*, *clec12b*, *cish,* and *znrf1*) in the high-18:3ω3 and high-18:2ω6 groups in the current study. However, *tlr5* showed significantly higher levels in the bacterin-injected high-18:2ω6 group than in the bacterin-injected high-18:3ω3 group, shedding light on the possible applications of high-ω6 diets to amplify pro-inflammatory signals, and high-ω3 diets to suppress inflammation.

On the other hand, the vectors EPA/ARA and DHA/ARA were plotted closer to the high-18:3ω3 group (Figure 6A). EPA and DHA [i.e., the anti-inflammatory FAs from the ω3 in “EPA/ARA” and “DHA/ARA”] induced changes in genes relevant to immune response, cell cycle, and apoptosis that may protect human THP-1 macrophages from an excessive inflammatory response (Allam-Ndoul et al., 2017). Both EPA/ARA and DHA/ARA were higher in the high-18:3ω3 group than in the high-18:2ω6 group (Table 3), which suggests a role for EPA and DHA (i.e., as 18:3ω3 elongation products and anti-inflammatory FAs) to possibly balance ARA in the high-18:3ω3 group for achieving homeostasis. Furthermore, *bcl3* (encoding protein with a putative role in inhibiting NF_K_B-mediated immune/inflammatory responses) was only induced within the high-18:3ω3 group, which might further indicate the specific anti-inflammatory effect of this high-ω3 diet.

It is noteworthy to mention that EPA/ARA and DGLA/ARA in the head kidney of the switched-diet salmon were not different from the high-18:3ω3 and high-18:2ω6 groups (Table 3), respectively. Also, (EPA + DGLA)/ARA and *15lox* loaded close to the switched-diet group in the PCoA (Figure 6A). Furthermore, *15lox* was one of the top contributors to dissimilarities between the high-18:3ω3 group and the switched-diet group (Figure 6C). Likewise, (EPA + DGLA)/ARA correlated positively with *15lox,* which is responsible for the production of specialized mediators that help resolve inflammation, as reviewed in the study of Snodgrass and Brüne (2019) (Figure 6B). A challenge in animal diets would be to formulate them with the benefit of the ω3-FAs (e.g., high EPA diets or promoting elongation from its ω3 precursor) without losing the beneficial effect generated by ω6 FAs (e.g., DGLA) (Nagy and Tiuca, 2017). It was previously reported that ω3-PUFA and ω6 gamma-linolenic acid may have synergistic anti-inflammatory effects (Balić et al., 2020). The fish of the switched-diet group may have benefited from the anti-inflammatory effects of DGLA and EPA to balance ARA. This was shown by the attenuated response of inflammatory relevant biomarkers, e.g., *ch25ha* and *znrf1* (Figures 2A, 4C), and a potentially reduced need for compensatory immunosuppressive processes as suggested by *clec12b* and *cish* (Figures 3B, 4B). The ω3 (i.e., EPA) and ω6 (i.e., DGLA) elongation products, during the change of tissue FA profile (evidenced in the switched-diet), might carry a possible solution to similar inflammatory conditions (e.g., pro-inflammatory responses associated with infection). There is growing evidence that some infection is associated with excessive pro-inflammatory responses that affect the normal function of several organs (Fajgenbaum and June, 2020). The present results (e.g., DGLA/ARA) suggest that the salmon elongated the available dietary FA precursors (e.g., 18:2ω6 in those fed the high-18:2ω6) in an attempt to balance the pro- and anti-inflammatory tissue FA profiles (e.g., DGLA in those fed the high-18:2ω6 group). However, the authors acknowledge the limitation of the current study to test the proposed hypothesis and recommend examining phospholipid FAs and quantifying the eicosanoids derived from those FAs in future studies.

## 5 Conclusion

Several of the *R. salmoninarum* bacterin-responsive biomarkers studied here did not show a significant response in the switched-diet group. While some transcripts with a putative role in the innate immunity antibacterial response (i.e., *ch25ha*, *clec12b*, *tlr5*, *cish*, and *znrf1*) were significantly induced by the bacterin only in the groups fed with high-18:3ω3 and high-18:2ω6 diets, other immune-relevant transcripts (i.e., *tnfrs6b*, *cish*, and *ccl13*) showed an antibacterial response in all dietary groups. This suggests a gene and pathway-specific effect of the tested immunomodulatory regimens. Fish in the high-18:2ω6 (a diet designed to be pro-inflammatory) and high-18:3ω3 (a diet designed to be anti-inflammatory) treatments showed a comparable antibacterial response overall, which suggests a compensatory mechanism for balancing immune responses through lipid metabolism (e.g., DGLA in those fed high-ω6) or negative feedback loops (e.g., *clec12b* and *cish* responses to the bacterin in high-18:2ω6 and high-18:3ω3) in these groups. DGLA and EPA (desaturation and elongation products of 18:2ω6 and 18:3ω3, respectively) may play major roles in balancing the ARA-derived pro-inflammatory effects. The switched-diet group exhibited an immunomodulatory effect through suppression of some components of the Atlantic salmon’s innate antibacterial response. The switched-diet approach may be suggested as a strategy for modulating Atlantic salmon antibacterial response and its associated pro-inflammatory status and minimizing immune response-related damage (in tissues) where needed. However, we acknowledge that the present results reflect the dietary FA-dependent response of Atlantic salmon to pathogen-derived antigens, and further investigations using live pathogens will be needed to determine if these immunomodulatory diets can enhance disease resistance in Atlantic salmon. Furthermore, we recommend that future research combine transcriptomics, lipidomics, and proteomics analyses to acquire a more complete picture of the molecular pathways and mechanisms involved in the dietary modulation of the anti-bacterin response.

## Data Availability

The datasets presented in this study can be found in online repositories. The names of the repository/repositories and accession number(s) can be found in the article/Supplementary Material.
