# Supplementary figures and images for: Nutritional immunomodulation of Atlantic salmon response to Renibacterium salmoninarum bacterin
Source: Front Mol Biosci. 2022 Sep 21;9:931548. doi: 10.3389/fmolb.2022.931548 (PMC9532746; doi:10.3389/fmolb.2022.931548)

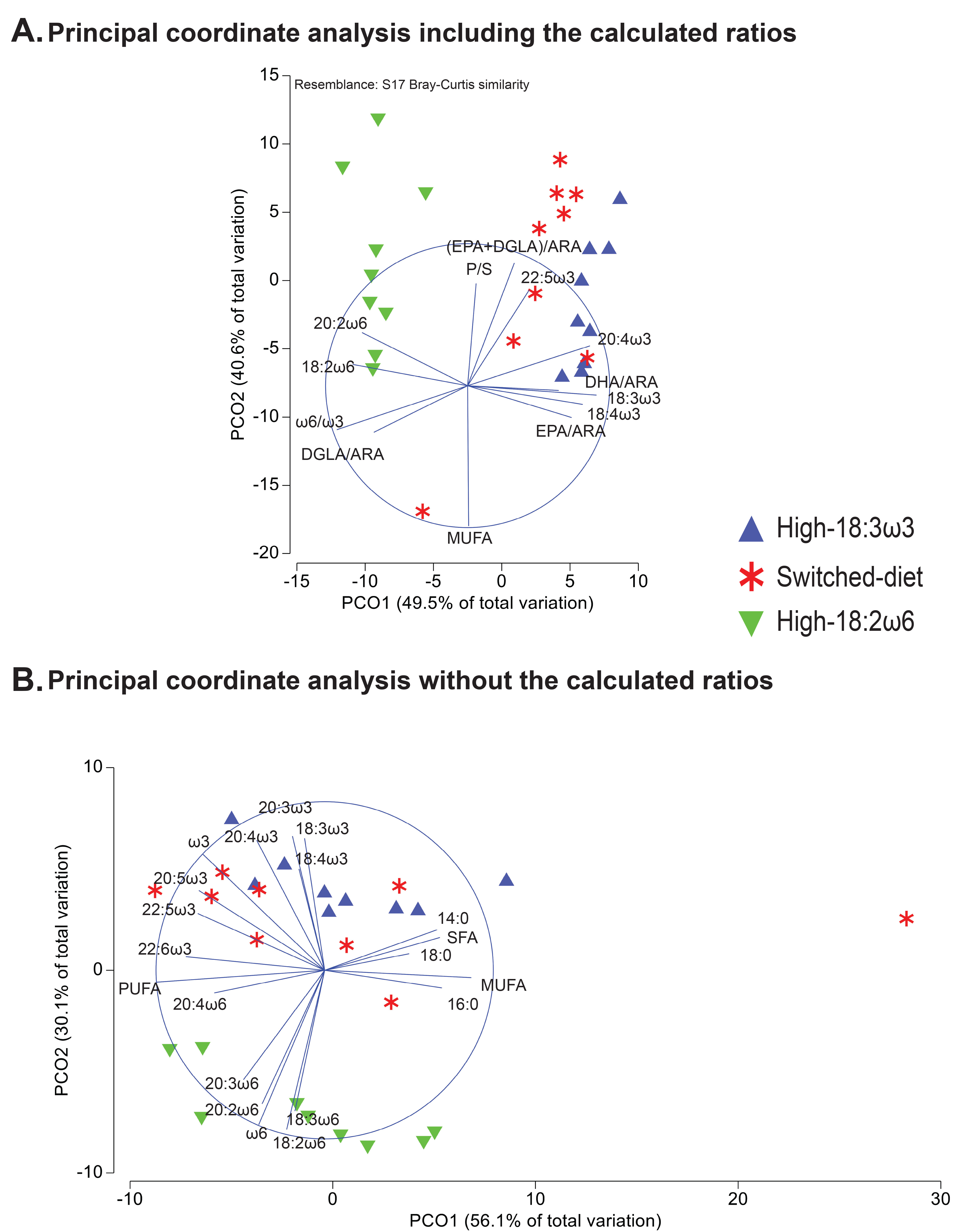

Supplement: Supplementary file 1 [file Image3.JPEG]

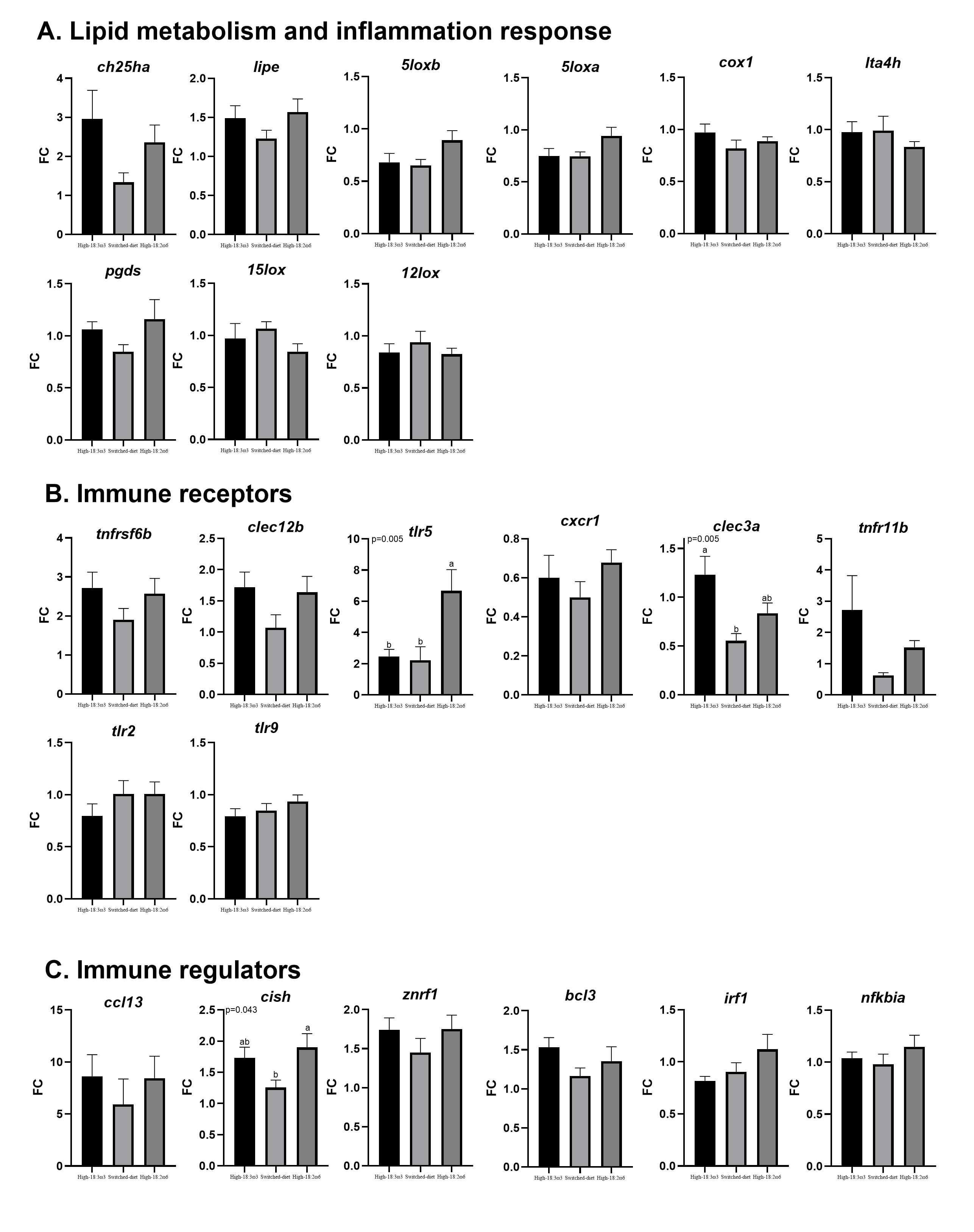

Supplement: Supplementary file 3 [file Image1.JPEG]

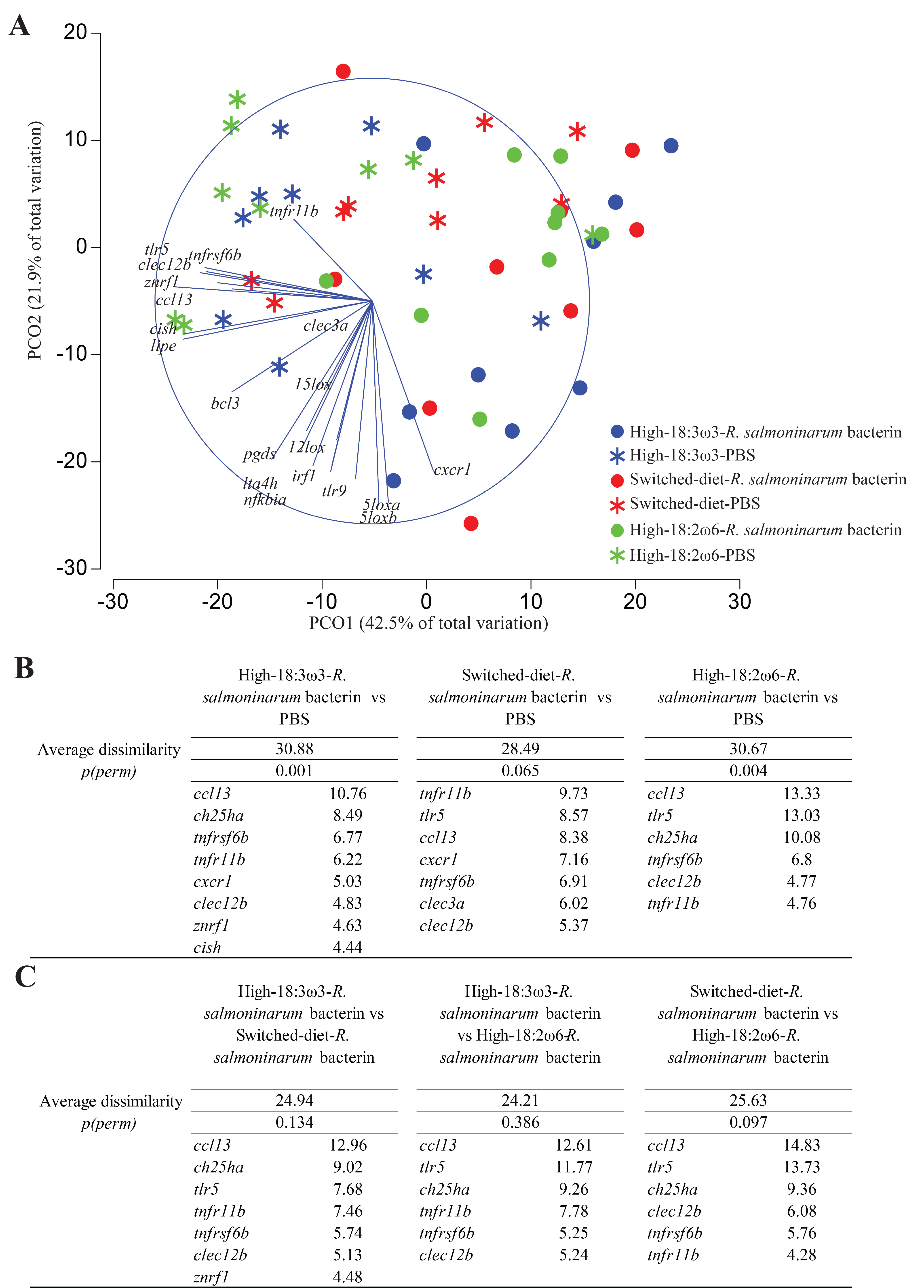

Supplement: Supplementary file 4 [file Image2.JPEG]
