# Supplementary material for: Nutritional immunomodulation of Atlantic salmon response to Renibacterium salmoninarum bacterin
Source: Front Mol Biosci. 2022 Sep 21;9:931548. doi: 10.3389/fmolb.2022.931548 (PMC9532746; doi:10.3389/fmolb.2022.931548)
Supplement: Supplementary file 2 [file Table1.docx]

**Table S.1** Relative quantity (RQ) of each qPCR-analyzed transcript

| Diet | High-18:3ω3 | | Switched-diet | | High-18:2ω6 | | one-way ANOVA | Two-way ANOVA | | |
| --- | --- | --- | --- | --- | --- | --- | --- | --- | --- | --- |
| Gene^1^\Injection | PBS | *R. salmoninarum*  bacterin | PBS | *R*. *salmoninarum* bacterin | PBS | *R. salmoninarum* bacterin |  | Injection | Diet | Interaction |
| *ch25ha †* | 5.76±1.88ᵇ | 17.01±4.24ᵃ | 5.51±1.23ᵇ | 7.36±1.33ᵇ | 7.18±3.78ᵇ | 16.97±3.15ᵃ | 0.01 | 0.002 | 0.277 | 0.200 |
| *lipe †* | 2.08±0.22ᵃᵇ | 3.1±0.33ᵃ | 1.82±0.15ᵇ | 2.24±0.2ᵃᵇ | 1.94±0.28ᵇ | 3.04±0.32ᵃ | p≤0.001 | p≤0.001 | 0.243 | 0.337 |
| *cox1* | 2.49±0.34 | 2.43±0.2 | 2.34±0.18 | 1.91±0.19 | 2.23±0.18 | 1.97±0.1 | 0.28 | 0.11 | 0.509 | 0.630 |
| *lta4h* | 2.13±0.22 | 2.08±0.22 | 1.95±0.21 | 1.93±0.27 | 2.16±0.28 | 1.8±0.11 | 0.86 | 0.411 | 0.852 | 0.67 |
| *pgds* | 1.88±0.31 | 2.01±0.14 | 2.02±0.27 | 1.71±0.14 | 1.78±0.15 | 2.07±0.34 | 0.87 | 0.864 | 0.962 | 0.414 |
| *15lox* | 2.39±0.28 | 2.32±0.34 | 1.88±0.23 | 2±0.13 | 2.23±0.17 | 1.88±0.17 | 0.46 | 0.581 | 0.425 | 0.576 |
| *12lox* | 2.12±0.26 | 1.78±0.18 | 1.81±0.2 | 1.7±0.19 | 2.08±0.17 | 1.71±0.12 | 0.46 | 0.065 | 0.768 | 0.73 |
| *5loxa* | 2.4±0.31 | 1.79±0.17 | 2.18±0.38 | 1.63±0.09 | 1.82±0.2 | 1.72±0.15 | 0.19 | 0.014 | 0.747 | 0.396 |
| *5loxb* | 2.99±0.42ᵃ | 2.03±0.26ᵇᶜ | 2.77±0.44ᵃᵇ | 1.8±0.16ᶜ | 2.05±0.16ᵇᶜ | 1.82±0.19ᶜ | 0.02 | 0.001 | 0.569 | 0.241 |
| *clec12b †* | 2.38±0.56 | 4.09±0.58 | 2.91±0.91 | 3.11±0.61 | 2.94±0.26 | 4.82±0.75 | 0.10 | 0.027 | 0.13 | 0.399 |
| *tnfrsf6b †* | 8.28±2.13ᵇ | 22.51±3.35ᵃ | 11.17±2.82ᵃᵇ | 21.27±3.22ᵃ | 8.48±1.63ᵇ | 21.82±3.32ᵃ | p≤0.001 | p≤0.001 | 0.723 | 0.768 |
| *tlr5 †* | 4.07±0.5ᵇ | 10.05±1.83ᵇ | 7.41±2.73ᵇ | 16.5±6.36ᵃᵇ | 3.8±0.84ᵇ | 25.37±5.15ᵃ | p≤0.001 | p≤0.001 | 0.151 | 0.091 |
| **clec3a* *†* | 3.28±0.49 | 4.05±0.61 | 5.46±1.09 | 3.03±0.41 | 4.26±0.96 | 3.57±0.46 | 0.21 | 0.723 | 0.187 | 0.093 |
| *tnfr11b †* | 3.27±0.72 | 8.86±3.61 | 11.11±5.31 | 6.95±0.93 | 5.61±1.33 | 8.48±1.26 | 0.44 | 0.522 | 0.636 | 0.193 |
| **cxcr1 †* | 5.89±0.74ᵃ | 3.54±0.68ᵃᵇ | 6.43±1.44ᵃ | 3.22±0.52ᵃᵇ | 3.66±0.36ᵃᵇ | 2.48±0.24ᵇ | 0.002 | 0.001 | 0.046 | 0.420 |
| *tlr2* | 2.31±0.31 | 1.85±0.26 | 1.92±0.32 | 1.93±0.25 | 2.3±0.32 | 2.31±0.27 | 0.69 | 0.533 | 0.504 | 0.624 |
| *tlr9* | 2.38±0.37 | 1.89±0.17 | 1.88±0.18 | 1.59±0.13 | 1.83±0.16 | 1.71±0.12 | 0.14 | 0.58 | 0.378 | 0.619 |
| *bcl3* | 1.96±0.24 | 3.01±0.24 | 2.12±0.44 | 2.47±0.22 | 2.09±0.29 | 2.82±0.39 | 0.12 | 0.007 | 0.864 | 0.527 |
| **ccl13 †* | 75.25±17.2ᵇ | 648.61±156.94ᵃ | 80.86±15.78ᵇ | 478.68±197.63ᵃᵇ | 92.84±26.06ᵇ | 783.8±196.2ᵃ | p≤0.001 | p≤0.001 | 0.487 | 0.536 |
| *cish †* | 1.78±0.22ᵇ | 3.09±0.31ᵃ | 1.79±0.23ᵇ | 2.25±0.21ᵃᵇ | 1.53±0.18ᵇ | 2.91±0.34ᵃ | p≤0.001 | p≤0.001 | 0.381 | 0.140 |
| *irf1* | 2.62±0.48 | 2.14±0.11 | 2.26±0.28 | 2.04±0.2 | 1.81±0.14 | 2.03±0.26 | 0.42 | 0.403 | 0.638 | 0.321 |
| *nfkbia* | 2.09±0.29 | 2.16±0.13 | 1.99±0.21 | 1.95±0.19 | 1.9±0.2 | 2.19±0.21 | 0.90 | 0.514 | 0.862 | 0.712 |
| *znrf1†* | 2.21±0.29ᵇ | 3.85±0.34ᵃ | 1.92±0.26ᵇ | 2.79±0.35ᵃᵇ | 2.07±0.28ᵇ | 3.62±0.37ᵃ | p≤0.001 | p≤0.001 | 0.119 | 0.431 |

^1^ Mean RQ ± SE (individual RQs used in the analysis n=9). Transcripts with asterisks “*” were analyzed using nonparametric statistics using SigmaPlot. Underlined are significantly different using T-test within the same dietary treatment. Different letters are significantly different using one-way ANOVA across all treatments. Gene symbol followed by dagger (†) were from (Eslamloo et al., 2020b).
