# Supplementary material for: Nutritional immunomodulation of Atlantic salmon response to Renibacterium salmoninarum bacterin
Source: Front Mol Biosci. 2022 Sep 21;9:931548. doi: 10.3389/fmolb.2022.931548 (PMC9532746; doi:10.3389/fmolb.2022.931548)
Supplement: Supplementary file 5 [file Table2.docx]

**Table S.2** Fatty acid composition (% total FAs^1^) to compare head kidney of Atlantic salmon fed high 18:2ω6 (i.e., before the dietary switch; tanks designated for the Switched-diet group) at week 4 and the Switched-diet at week 8

| FA composition (% total FAs) | High-18:2ω6 (week 4, before diet switch) | Switched-diet | p-value^6^ |
| --- | --- | --- | --- |
| 14:0 | 1.79±0.14 | 1.43±0.13 | 0.074 |
| 16:0 | 14.42±0.27 | 15.5±1.37 | 0.452 |
| 18:0 | 4.87±0.19 | 5.1±0.44 | 0.648 |
| 18:2ω6 | 15.99±0.55 | 13.75±0.81 | **0.036** |
| 20:3ω6 (DGLA) | 1.63±0.16 | 1.16±0.1 | **0.026** |
| 20:4ω6 (ARA) | 2.61±0.28 | 2.01±0.29 | 0.151 |
| 18:3ω3 | 1.99±0.09 | 6.39±0.56 | **<0.001** |
| 18:4ω3 | 0.85±0.05 | 1.35±0.16 | **0.009** |
| 20:4ω3 | 0.58±0.03 | 1.11±0.1 | **<0.001** |
| 20:5ω3 (EPA) | 3.67±0.19 | 3.38±0.39 | 0.505 |
| 22:5ω3 | 1.09±0.06 | 0.98±0.1 | 0.365 |
| 22:6ω3 (DHA) | 11.36±0.81 | 10.06±1.45 | 0.444 |
| ΣSFA^2^ | 22.13±0.36 | 23.12±1.8 | 0.596 |
| ΣMUFA^3^ | 34.77±1.5 | 33.57±1.93 | 0.631 |
| ΣPUFA^4^ | 42.92±1.25 | 43.2±3.3 | 0.938 |
| P/S^5^ | 1.94±0.04 | 1.98±0.19 | 0.830 |
| Σω3 | 19.88±0.84 | 23.93±2.48 | 0.142 |
| DHA/EPA | 3.1±0.14 | 2.93±0.16 | 0.433 |
| Σω6 | 22.23±0.76 | 18.56±1.01 | **0.010** |
| ω6/ω3 | 1.13±0.05 | 0.88±0.13 | 0.080 |
| EPA/ARA | 1.49±0.12 | 1.79±0.2 | 0.218 |
| DHA/ARA | 4.5±0.24 | 5.05±0.39 | 0.249 |
| DGLA/ARA | 0.64±0.03 | 0.65±0.07 | 0.856 |
| EPA+DGLA/ARA | 3.12±0.09 | 2.95±0.2 | 0.455 |

^1^ mean ± s.e. (n = 9).

^2^ saturated fatty acids.

^3^ monounsaturated fatty acids.

^4^ polyunsaturated fatty acids.

^5^ polyunsaturated fatty acids/ saturated fatty acids

^6^ p-values that were <0.05 (i.e., significant) were bolded.
